# MicroRNA-193b impairs muscle growth in mouse models of type 2 diabetes by targeting the PDK1/Akt signalling pathway

**DOI:** 10.1007/s00125-021-05616-y

**Published:** 2021-12-16

**Authors:** Shu Yang, Guangyan Yang, Han Wu, Lin Kang, Jiaqing Xiang, Peilin Zheng, Shanhu Qiu, Zhen Liang, Yan Lu, Lijing Jia

**Affiliations:** 1 Department of Endocrinology, The Second Clinical Medical College, Jinan University (Shenzhen People’s Hospital), Shenzhen, China; 2 Integrated Chinese and Western Medicine Postdoctoral Research Station, Jinan University, Guangzhou, China; 3 Department of Endocrinology, Key Laboratory of Endocrinology, National Health Commission, Peking Union Medical College Hospital, Peking Union Medical College and Chinese Academy of Medical Sciences, Beijing, China; 4 Department of Geriatrics, The Second Clinical Medical College, Jinan University (Shenzhen People’s Hospital), Shenzhen, China; 5 Department of Endocrinology and Metabolism, Zhongshan Hospital, Fudan University, Shanghai, China

**Keywords:** Akt, miR-193b, Muscles atrophy, PDK1, Type 2 diabetes

## Abstract

**Aims/hypothesis:**

Type 2 diabetes is associated with a reduction in skeletal muscle mass; however, how the progression of sarcopenia is induced and regulated remains largely unknown. We aimed to find out whether a specific microRNA (miR) may contribute to skeletal muscle atrophy in type 2 diabetes.

**Methods:**

Adeno-associated virus (AAV)-mediated skeletal muscle miR-193b overexpression in C57BLKS/J mice, and skeletal muscle miR-193b deficiency in *db/db* mice were used to explore the function of miR-193b in muscle loss. In C57BL/6 J mice, tibialis anterior-specific deletion of 3-phosphoinositide-dependent protein kinase-1 (PDK1), mediated by in situ AAV injection, was used to confirm whether miR-193b regulates muscle growth through PDK1. Serum miR-193b levels were also analysed in healthy individuals (*n* = 20) and those with type 2 diabetes (*n* = 20), and correlations of miR-193b levels with HbA_1c_, fasting blood glucose (FBG), body composition, triacylglycerols and C-peptide were assessed.

**Results:**

In this study, we found that serum miR-193b levels increased in individuals with type 2 diabetes and negatively correlated with muscle mass in these participants. Functional studies further showed that AAV-mediated overexpression of miR-193b induced muscle loss and dysfunction in healthy mice. In contrast, suppression of miR-193b attenuated muscle loss and dysfunction in *db/db* mice. Mechanistic analysis revealed that miR-193b could target *Pdk1* expression to inactivate the Akt/mammalian target of rapamycin (mTOR)/p70S6 kinase (S6K) pathway, thereby inhibiting protein synthesis. Therefore, knockdown of PDK1 in healthy mice blocked miR-193b-induced inactivation of the Akt/mTOR/S6K pathway and impairment of muscle growth.

**Conclusions/interpretation:**

Our results identified a previously unrecognised role of miR-193b in muscle function and mass that could be a potential therapeutic target for treating sarcopenia.

**Graphical abstract:**

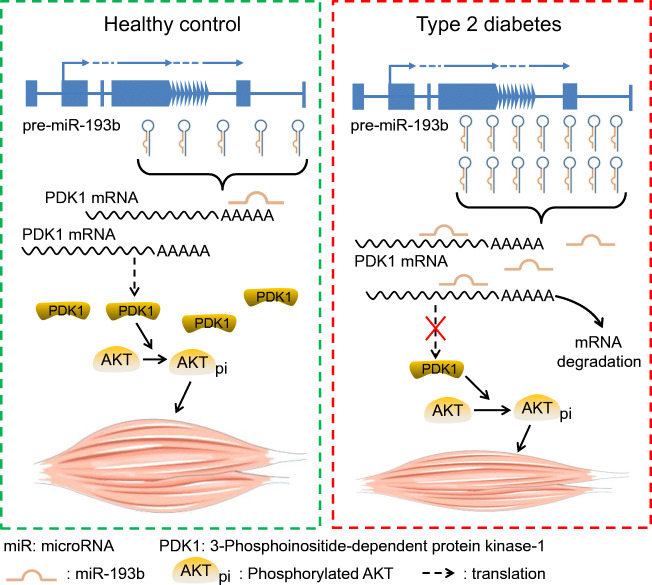

**Supplementary Information:**

The online version contains peer-reviewed but unedited supplementary material available at 10.1007/s00125-021-05616-y.

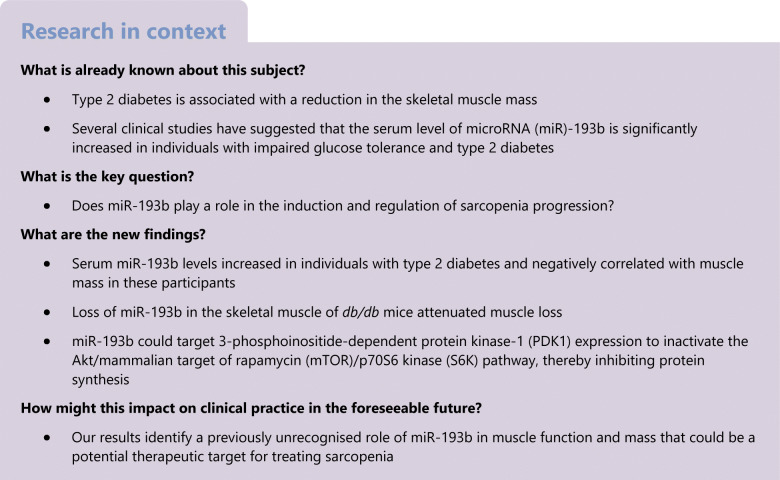



## Introduction

With the development of the social economy and changes in lifestyle, the incidence of metabolic disorders, such as type 2 diabetes, has increased significantly every year in the past few decades [1–3]. According to the International Diabetes Federation Diabetes Atlas, 9th edition, approximately 463 million adults aged between 20 and 79 years were estimated to have diabetes in 2019, globally, i.e. one out of 11 adults were estimated to have diabetes. Diabetes is expected to hit 578.4 million people by 2030 and 702 million by 2045.

Sarcopenia, the degenerative loss of skeletal muscle mass, is related to frailty and geriatric syndromes, and can affect the quality of life in elderly people and even cause death in severe cases [4, 5]. Unfortunately, there are no effective pharmacological treatments for this devastating disease. Sarcopenia is believed to be closely associated with the metabolic syndrome, especially type 2 diabetes. Studies show that type 2 diabetes not only accelerates the degeneration of muscle mass and strength, but that the rate of degeneration also increases with diabetes duration and elevated HbA_1c_ levels [6, 7]. Likewise, the risk of sarcopenia is significantly higher in individuals with type 2 diabetes than in those without (~15.7% vs ~6.9%) [8]. However, the molecular mechanism by which type 2 diabetes accelerates the progression of sarcopenia remains unclear, and this has greatly hindered the development of therapeutic methods for type 2 diabetes-associated muscle atrophy.

Chronic insulin resistance is an important driving force for muscle atrophy in type 2 diabetes as insulin resistance could lead to reduced protein synthesis and increased protein degradation [9]. Classical insulin signalling pathways and anabolic stimuli, including phosphatidylinositol 3-kinase (PI3K), 3-phosphoinositide-dependent protein kinase-1 (PDK1), Akt, mammalian target of rapamycin (mTOR) and p70S6 kinase (S6K), activate protein synthesis, thereby contributing to muscle growth [10–12]. Consistent with this, the genetic activation of IGF1 or Akt in skeletal muscle is sufficient to increase muscle mass [11]. IGF1 plays a central role in the anabolic signalling pathway by activating PDK1. Subsequently, PDK1 activates the Akt/mTOR/S6K pathway, which in turn promotes protein synthesis while suppressing protein degradation by inhibiting the forkhead box protein O1 (FOXO1) transcription factor, which regulates the muscle-specific ubiquitin ligases, muscle really interesting new gene (RING)-finger protein-1 (MuRF1) and Atrogin-1 [13]. Notably, previous research has demonstrated the effects of indoprofen in boosting skeletal muscle mass through the sequential activation of PDK1/Akt/S6K and AMP-activated protein kinase (AMPK)/peroxisome proliferator-activated receptor, gamma, coactivator 1, alpha (PGC-1α), suggesting that PDK1 is an effective target for improving muscle atrophy [10].

MicroRNAs (miRs) are a class of non-coding single-stranded RNA molecules with a length of approximately 22 nucleotides, encoded by endogenous genes. Growing evidence indicates that miRs are involved in the regulation of skeletal muscle. Furthermore, evidence suggests that muscle-expressed miR-23a/27a can ameliorate type 2 diabetes-induced muscle atrophy by increasing skeletal muscle insulin sensitivity [14]. The long non-coding (lnc)RNA-IRS1 activates the IGF1/PI3K/Akt signalling pathway through the adsorption of miR-15 to improve insulin sensitivity and promote muscle regeneration [15]. A subset of miRs in the *Dlk1*-*Dio3* cluster regulates age-associated muscle atrophy by targeting Atrogin-1 [16]. This suggests that miRs targeting Akt-signalling-pathway-related genes could be effective in improving muscle atrophy. However, the key miRs that contribute to skeletal muscle atrophy in individuals with type 2 diabetes need to be explored further.

## Methods

### Studies in animals

All animal care and experimental protocols for in vivo studies conformed to the Guide for the Care and Use of Laboratory Animals, published by the National Institutes of Health (NIH; NIH publication no.: 85–23, revised 1996), and were approved by the Ethics Committee of the Second Clinical Medical College of Jinan University, Shenzhen People’s Hospital (Shenzhen, China). All animals were randomised before they received treatment.

Male mouse models of type 2 diabetes (BKS.C g–m +/+ Leprdb/J [referred to herein as *db/db* mice]; 8 ± 0.5 weeks old; 42 ± 3 g) and male C57BLKS/J wild-type mice (C57 mice; 8 ± 0.5 weeks old; 24 ± 1 g) were purchased from the Animal Center of Nanjing University (Nanjing, Jiangsu, China). These mice were maintained at the Animal Center of Shenzhen People’s Hospital (Shenzhen, China) with free access to food and drinking water. Up to five mice were kept per plastic cage with corn cob bedding material. In addition, we administered treatment to mice in a blinded fashion.

### High-fat diet feeding of mice

C57 mice (8 ± 0.5 weeks old; 24 ± 1 g) were randomly assigned into four groups of *n* = 5 animals and fed with a control diet (catalogue no.: D12052701M; Research Diets, New Brunswick, NJ, USA) or high-fat diet (HFD; catalogue no.: D12492; Research Diets) for 16 weeks. After treatment, all mice were anaesthetised and euthanised in a CO_2_ chamber following 16 h of fasting, and blood samples and muscles were harvested for further analysis.

### In situ treatment of tibialis anterior with adeno-associated virus in C57 mice

For in vivo skeletal muscle miR-193b overexpression, adeno-associated virus (AAV)9-human α-skeletal actin (hsa)-miR-193b (0.5–1.5 × 10^11^ viral genomes [vg]/ml in 50 μl saline [0.15 mol/l NaCl]; GeneChem Company, Shanghai, China) was injected into tibialis anterior (TA) from C57 mice (8 ± 0.5 weeks old; 24 ± 1 g) to induce TA miR-193b overexpression (*n* = 5 mice). After 8 weeks of treatment, all mice were anaesthetised and euthanised in a CO_2_ chamber, and then blood and tissue samples were obtained for further analysis.

### Systemic injection of AAV9-hsa-miR-193b in C57 mice

AAV9-hsa-miR-193b (GeneChem Company) or AAV9-hsa-*gfp* (0.5–1.5 × 10^12^ vg/ml in 200 μl saline [0.15 mol/l NaCl]; GeneChem Company) was injected into the tail vein of C57 mice to induce miR-193b overexpression in mice (*n* = 5 per group). During treatment, the body weight of mice was determined weekly and average food intake was recorded daily. In addition, GTTs and ITTs were performed. After 8 weeks of treatment, mice underwent skeletal muscle performance and endurance tests, and refed blood glucose levels and body composition were measured (see below). Subsequently, all mice were fasted overnight (16 h), anaesthetised and euthanised in a CO_2_ chamber, and then blood and tissue samples were obtained for further analysis. Tibia bones were isolated from mice, hair was rinsed off and then bone length was measured using a ruler. Whole hearts from mice were weighed and normalised to tibia length.

### Induction of miR-193b-deficiency in C57

sgmiR-193b AAV or AAV9-hsa-*gfp* (0.5–1.5 × 10^12^ vg/ml in 200 μl saline [0.15 mol/l NaCl]) was injected into the tail vein of C57 mice to result in miR-193b-deficient C57 mice (*n* = 5 per group). During treatment, the body weight of mice determined weekly. After 8 weeks of treatment, body composition measurements were performed on all mice (see below). Subsequently, mice were fasted overnight, anaesthetised and euthanised in a CO_2_ chamber, and then blood samples and tissues were collected for further analysis.

### Induction of miR-193b deficiency in *db*/*db* mice

AAV9-hsa-miR-193b-sponge (sgmiR-193b AAV; GeneChem Company) or AAV9-hsa-*gfp* (0.5–1.5 × 10^12^ vg/ml in 200 μl saline [0.15 mol/l NaCl]) was injected into the tail vein of *db/db* mice to result in miR-193b-deficient *db/db* mice (*n* = 5 per group). During treatment, body weight of mice was determined weekly and average food intake was recorded daily. GTTs and ITTs were performed as described below. After 8 weeks of treatment, body composition measurements were performed on all mice, as well as skeletal muscle performance and endurance tests (see below), and refed blood glucose analysis. Subsequently, mice were fasted overnight (16 h), anaesthetised and euthanised in a CO_2_ chamber, and then blood samples and tissues were collected for further analysis. Tibia bones were isolated from mice, hair was rinsed off and then bone length was measured using a ruler. Whole hearts from mice were weighed and were normalised to tibia length.

### PDK1 knockdown in skeletal muscle of miR-193b overexpressing mice

To induce skeletal muscle PDK1 knockdown in miR-193b overexpressing mice, AAV9-hsa-short hairpin (sh)*Pdk1* (PDK1 knockdown group; GeneChem Company) or AAV9-hsa-*gfp* (control group) was pre-injected into the TA muscle of C57 mice three times at three independent locations (*n* = 5 per group) and, after 2 weeks, AAV9-hsa-miR-193b or AAV9-hsa-*gfp* (0.5–1.5 × 10^11^ vg/mL in 50 μl saline [0.15 mol/l NaCl]) was injected into the TA muscle at three independent locations. After 4 weeks of treatment with AAV9-hsa-miR-193b or AAV9-hsa-*gfp*, all mice were fasted overnight and then were anaesthetised and euthanised in a CO_2_ chamber, after which blood samples and tissues were procured for further analysis (see below).

### miR-sequencing

miRs were extracted from TA muscle from 16-week-old male C57 and *db/db* mice (*n* = 4 per group) using a Trizol reagent kit (Invitrogen, Carlsbad, CA, USA) and analysed by Illumina HiSeq Xten microarray (Illumina, CA, USA) by Gene Denovo Biotechnology (Guangzhou, China). Differential expression of miRs between two different groups was analysed using edgeR software (version 3.12.1; www.bioconductor.org/packages/3.2/bioc/html/edgeR.html) [17]. miRs with a *p* value <0.05 were considered as being significantly differentially expressed. The Wilcoxon signed rank test, a commonly utilised sub-parameter test, was chosen for the calculation of *p* values for miRs sequencing. A two-tailed unpaired Student’s *t* test was used for comparisons between two groups.

### EdgeR methodology

EdgeR is based on the principles of the negative binomial distribution model for sequencing data, as follows: (1) input data with matrix row names sampled and column names miRs; (2) set grouping information and remove low-expression miRs while normalising; (3) apply quantile-adjusted conditional maximum likelihood (qCML) for dispersion estimation; and (4) calculate differential genes. For calculation of fold values, first the expression matrix was log-transformed (log_2_) and, subsequently, the difference between the mean expression of the case and control groups was log-transformed (log_2_) and presented as fold change, calculated as follows: log_2_(*x*/*y*) = log_2_(*x*) − log_2_(*y*), where *x* and *y* represent the mean expression of miRs in the representative case and control groups, respectively. Positive values represent increases in miR expression and negative values represent decreases in miR expression.

### Analysis of miR-193b levels

An miR-193b primer (Tsingke, Beijing, China) was used, as described in previous studies [18, 19] to determine miR-193b levels in mouse (untreated C57 and *db/db*; HFD-fed C57; miR-193b AAV-treated C57; sgmiR-193b AAV-treated *db*/*db* and C57) tissue (TA, gastrocnemius muscle, soleus muscle, heart, forebrain, cerebellum or liver) and serum samples. Serum miR-193b levels were normalised to cel-miR-39 (Invitrogen) and tissue miR-193b levels were normalised to the mRNA level of β-actin (see electronic supplementary material [ESM] Methods for further details).

### H&E staining

TA and gastrocnemius muscle tissue from AAV-treated mice/tissues (miR-193b AAV-treated C57; miR-193b AAV-treated TA from C57; sgmiR-193b AAV-treated C57 or *db*/*db*; sh*Pdk1* AAV-treated C57; sh*Pdk1* AAV + miR-193b AAV-treated C57) and their controls were weighed and subjected to H&E staining (see ESM Methods for further details).

### Body composition measurements

Total fat and lean mass were analysed using a fully automatic EchoMRI system (Echo Medical Systems, TX, USA) according to the manufacturers’ instructions [20].

### Determination of serum glucose and insulin levels

Serum samples from miR-193b AAV-treated C57 and AAV-hsa-sgmiR-193b-treated *db*/*db* mice were used to measure blood glucose and insulin levels via OneTouch glucometer and test strips (LifeScan) or an insulin ELISA kit (catalogue no.: ab277390; Abcam, Cambridge, MA, USA), respectively (see ESM Methods). For refed glucose samples, miR-193b AAV-treated C57 and sgmiR-193b AAV-treated *db*/*db* mice (and their controls) were fasted overnight and then food was replaced for 2 h before determination of refed blood glucose (see ESM Methods).

### GTT and ITT

GTTs and ITTs were performed in mice at 15 and 16 weeks of age. Glucose levels were determined at 0, 15, 30, 60, 90 and 120 min after injection using OneTouch glucometer and test strips (LifeScan, Milpitas, CA, USA) (see ESM Methods for further details).

### Immunofluorescent staining for myofibre type

Immunofluorescent staining was conducted for myofibre type in gastrocnemius muscle from miR-193b AAV-treated C57 and sgmiR-193b AAV-treated *db*/*db* mice (see ESM Methods). In brief, gastrocnemius muscle and TA slices were subjected to myofibre-type staining using the Muscle Fiber Typing Staining Kit (Servicebio, Wuhan, China), as per manufacturer’s instructions. Rabbit anti-fast myosin skeletal heavy chain antibody (diluted 1:100 in PBS; catalogue no.: GB112130; Servicebio) and mouse anti-slow skeletal myosin heavy chain antibody (diluted 1:100 in PBS; catalogue no.: GB21303; Servicebio) were used as primary antibodies, while Cy3-labelled goat anti-rabbit IgG (catalogue no.: GB21303; Servicebio) and Alexa Fluor 488-labelled goat anti-mouse IgG (catalogue no.: GB25301; Servicebio) (both diluted 1:400) were used as secondary antibodies. DAPI (catalogue no.: P0131-25 ml; Beyotime, Shanghai, China) was used to stain cell nuclei. Images were captured and processed with a Leica DMi8 automated microscope (Leica, Weztlar, Germany) and the myofibre area was measured using ImageJ software (RRID: SCR_003070; version 1.51; NIH, Bethesda, MD, USA) (see ESM Methods for further details).

### Quantitative real-time PCR analysis

Briefly, for quantitative real-time PCR (qPCR), total RNA was extracted from mouse muscle tissue (miR-193b AAV-treated C57; miR-193b AAV-treated TA from C57; sgmiR-193b AAV-treated C57 or *db*/*db*; sh*Pdk1* AAV-treated C57; sh*Pdk1* AAV + miR-193b AAV-treated C57) using RNAiso Plus (Sigma-Aldrich, St Louis, MO, USA), as reported previously [21]. mRNA expression was normalised to the β-actin by Quantity One Software (Bio-Rad Laboratory, CA, USA) (see ESM Methods for full details and ESM Table 1 for a list of primers).

### Mitochondrial DNA analysis

Mitochondrial DNA (mtDNA) copy number was determined in muscle tissue from mice (miR-193b AAV-treated C57; sgmiR-193b AAV-treated *db*/*db* or C57) by PCR. mtDNA or nuclear DNA was amplified using primers against *Cox-3* or *Act1*, respectively (see ESM Methods).

### Analysis of glycogen and lactate content

Glycogen and lactate content in mouse skeletal muscle (miR-193b AAV-treated C57; sgmiR-193b AAV-treated *db*/*db*) was assessed using a glycogen assay kit (catalogue no.: ab65620, Abcam) and a lactated assay kit (catalogue no.: ab169558, Abcam), as per manufacturer’s instructions (see ESM Methods).

### Analysis of skeletal muscle performance and endurance

Hanging time, locomotor activity, grip strength and maximal oxygen consumption were assessed in all mice after 8 weeks of AAV treatment (see ESM Methods for details). In brief, for the hanging test, mice were placed on a wire length and the wire was rotated so that mice were hanging. Hanging time was recorded. To assess locomotor activity, a computer-controlled, open-circuit system (Oxymax Comprehensive Lab Animal Monitoring System; Columbus Instruments, Columbus, OH, USA) was used. Forelimb grip strength was assessed using a digital grip strength meter (Columbus Instruments). To do this, mice were lifted by their tails, allowed to grasp the forelimb pull bar and pulled smoothly away from the transducer in the horizontal plane. Maximum force exerted on the transducer was recorded. To assess maximal oxygen consumption, mice were placed on the treadmill (Jiangsu SANS Biological Technology, China) and the shock grid was activated. Treadmill speeds were then increased until exhaustion.

### Western blotting

Protein lysates were prepared from mouse muscle samples (miR-193b AAV-treated C57; sgmiR-193b AAV-treated *db*/*db*; sh*Pdk1* AAV-treated C57; sh*Pdk1* AAV + miR-193b AAV-treated C57), as previously described [22], and incubated with antibodies against β-actin, S6K, mTOR, p-mTOR, Akt, p-Akt and myosin heavy chain 7B (Myh) (all from Abcam); and p-S6K (Thr389), PDK1, AMPKα, and p-AMPKα (all from Cell Signaling Technology, MA, USA) (see ESM Methods for details). Densitometry analysis was performed using Quantity One Software (Bio-Rad Laboratory) and protein levels were normalised to β-actin.

### Collection and analysis of clinical samples

The basic clinical characteristics of human participants are shown in ESM Table 2 and ESM Table 3. Venous blood samples were collected from *n* = 20 individuals with type 2 diabetes and *n* = 20 healthy control participants after an overnight (≥10 h) fast. Concentrations of fasting blood glucose (FBG) and triacylglycerol were measured with an automatic biochemical analyser (AU5800; Beckman Coulter, USA). Serum C-peptide levels were measured by direct chemiluminescence immunoassay (SIMENS ADVIA Centaur XP, Germany). HbA_1c_ was measured using a dedicated high-performance liquid chromatography system (catalogue no.: D100; Bio-Rad Laboratory). Reference ranges were obtained from the central laboratory of Shenzhen People’s Hospital (Shenzhen, China) and the variables measured all sat within references ranges based on age, sex and ethnicity. Body compositions were measured via a bioelectrical impedance device (catalogue no.: INBODY770; InBody, Shanghai, China).

In addition, qPCR was performed to detect miR-193b content in serum (see above and ESM Methods).

This work was approved by the Institutional Review Board and the Ethics Committee of the Shenzhen People’s Hospital (approval no. LL-KT-2018338). Informed written consent was obtained from all participants prior to inclusion in the study.

### Cell culture

C2C12 myoblasts (catalogue no.: SCSP-505) were purchased from the National Collection of Authenticated Cell Cultures (Shanghai, China). Cells were regularly checked for mycoplasma in a standardised manner, by a qPCR test, performed under ISO17025 accreditation to ensure work was conducted in mycoplasma-negative cells. C2C12 myoblasts were maintained as previously described [23]. They were cultured in growth medium (Dulbecco’s Modified Eagle Medium, high glucose; Gibco, Grand Island, NY, USA) containing 10% (vol./vol.) fetal bovine serum (Gibco), 10 U/ml penicillin and 10 μg/ml streptomycin (Welgene, Taipei, China), at 37°C, 5% CO_2_. Differentiation medium contained Dulbecco’s Modified Eagle Medium, high glucose, and 2% (vol./vol.) horse serum, 10 U/ml of penicillin and 10 μg/ml of streptomycin. Before treatment, C2C12 cells were cultured in normal differentiation medium for 1 day.

### Predicted targets of miR-193b

StarBase (http://starbase.sysu.edu.cn/, accessed 5 January 2020) was used to predicted targets of miR-193b. In the query page of miRNA–mRNA interactions, we entered ‘miR-193b’ into the microRNA search bar, resulting in the predicted targets of miR-193b being displayed.

### C2C12 treatment and miR-193b mimic/inhibitor transient transfection

C2C12 cells were treated with dexamethasone (DEX; catalogue no.: HY-N2609) and TNF-α (catalogue no.: HY-P1825), both obtained from MedChemExpress (Monmouth Junction, NJ, USA), to induce insulin resistance and inflammation, respectively. All cell experiments were conducted in a blinded fashion.

miR-193b mimic/inhibitor transient transfections were also conducted in C2C12 cells. miR-193b mimic and inhibitor sequences have been described in a previous study [24]. The miR-193b mimic was a duplex RNA, with the sense sequence 5′-aacuggcccacaaaguccc-3′ and the antisense sequence 5′-gacuuugugggccaguuuu-3′. Nontargeting negative control sequences (sense 5′-uucuccgaacgugucacgutt-3′, antisense 5′-acgugacacguucggagaatt-3′) were used as controls. The inhibitor of miR-193b (5′-gggacuuugugggccaguu-3′) was a single RNA sequence that was exactly complementary to miR-193b. A nontargeting negative control sequence (5′-caguacuuuuguguaguacaa-3′) was used as a control. Transient transfections were performed using Lipofectamine 3000 (Invitrogen), according to the protocol provided by the manufacturers.

After treatment, cells were harvested for myosin immunostaining (see below), or PCR analysis of miR-193b, *PDK1* and *Dnmt1* expression, and western blotting for analysis of PDK1, Akt, p-Akt, Myh, mTOR and p-mTOR content, as described above (see ESM Methods and ESM Table 1).

### Immunofluorescent staining of C2C12 cells

Myofibre immunostaining of C2C12 cells was performed using primary antibodies against Myh (catalogue no.: ab172967; Abcam). Staining for muscle fibre-type was performed using the Muscle Fiber Typing Staining Kit (Servicebio), as per manufacturer’s instructions. Images were captured and processed with a Leica DMi8 automated microscope (Leica), and the myofibre area was measured using ImageJ software (RRID: SCR_003070; version 1.51; NIH) (see ESM Methods).

### Dual luciferase reporter assay

C2C12 cells were transfected with 3′ untranslated region (UTR) luciferase reporter constructs (*Pdk1* 3′-UTR or *Pdk1* 3′-UTR-mutant), miRNA (control RNA [catalogue no.: sc-36,869; Santa Cruz Biotechnology] or miR-193b-3p) and *Renilla* luciferase using Lipofectamine 3000 (Invitrogen). After 48 h, luciferase activity was measured using a Dual Luciferase Reporter Assay Kit (catalogue no.: E1910; Promega, Madison, WI, USA) and microplate reader (catalogue no.: H1; Biotek, Winooski, VT, USA). *Renilla* luciferase was used to normalise values (see ESM Methods).

### Small interfering RNA targeting of *Pdk1* in C2C12 cells

Small interfering RNA (siRNA) against *Pdk1* (catalogue no.: sc-36,242) and scrambled siRNA were purchased from Santa Cruz Biotechnology (CA, USA). Details of *Pdk1* siRNA resuspension can be found in the ESM Methods. C2C12 cells were transfected with scrambled or target siRNA (100 nmol/l) using Lipofectamine 3000 (Invitrogen). After 6 h of transfection, the cells were added to an equivalent volume of medium and transfection was continued for 24 h. Following this, cells were treated with miR-193b inhibitor or nontargeting negative control RNA and western blot analysis was used to determine the protein level of PDK1, Akt and p-Akt (see ESM Methods).

### Human dataset analysis

Global gene expression profiles of skeletal muscle in male individuals with type 2 diabetes were acquired from the Gene Expression Omnibus (GEO) GSE29221 dataset of the National Center for Biotechnology Information (NCBI; www.ncbi.nlm.nih.gov/gds, accessed 9 June 2020). The GEO2R online analysis tool (www.ncbi.nlm.nih.gov/geo/geo2r/; accessed 9 June 2020) was used to analyse *DNMT1* expression in skeletal muscle samples from individuals with and without diabetes.

### Data analysis

All the data were generated from at least three independent experiments. No data, sample cells or mice were excluded from statistical analysis. Data are expressed as mean ± SD of each group. Based on our previous studies and/or preliminary experiments, we calculated the group size required for in vivo and in vitro studies. The density of images was quantified by an individual who was blinded to the treatment, using ImageJ software (RRID: SCR_003070; version 1.51; NIH). The density of each target band was normalised to β-actin in the corresponding sample to reduce variance. qPCR data and western blot quantifications were expressed as fold of the control group’s mean value, which was defined as 1 plus its own SD. A two-tailed unpaired Student’s *t* test was used for comparison between two groups. Comparisons between more than two experimental groups were conducted by one-way ANOVA, followed by Bonferroni’s post hoc test. All analyses were carried out using GraphPad Prism 7.04 (www.graphpad.com/). *p* < 0.05 was considered statistically significant.

## Results

### Circulating miR-193b increased in *db/db* mice and correlated negatively with muscle mass in these mice

**Fig. 1:**
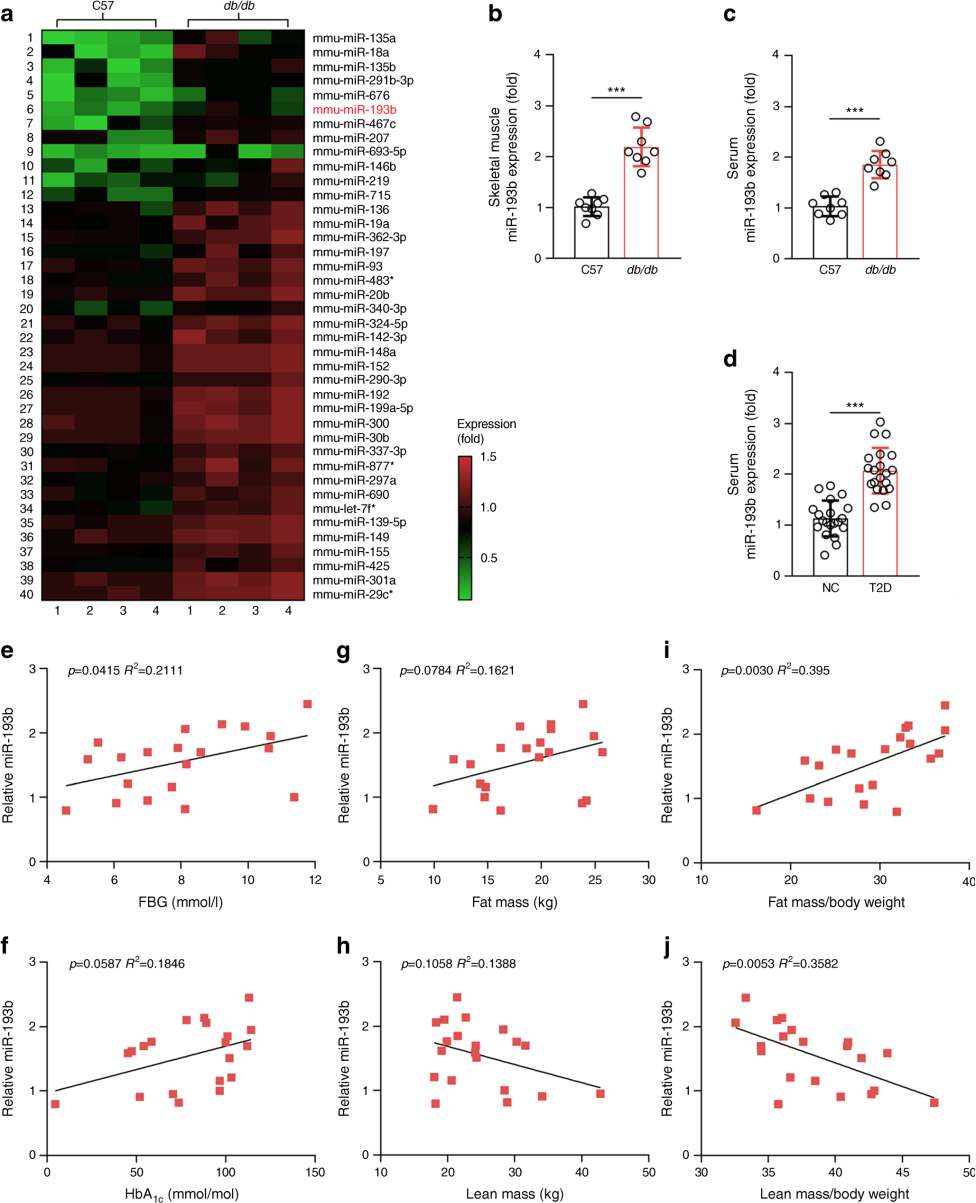
miR-193b is negatively correlated with lean mass. (**a**) miR expression in TA of C57 mice and *db/db* mice. All miRs presented are significantly different vs C57 mice (*p* < 0.05, analysed by Wilcoxon signed rank test; only the top 40 miRs are shown; *n* = 4). miRs are sorted by fold change, shown in descending order from top to bottom. The asterisks (*) next to miR names in (**a**) indicate that the miR comes from the opposite arm of the precursor. mmu, *Mus musculus*. (**b**, **c**) miR-193b expression in skeletal muscle (**b**) and serum (**c**) of C57 and *db/db* mice (*n* = 8). (**d**) miR-193b levels in serum of healthy control human participants (*n* = 20) and of individuals with type 2 diabetes (T2D; *n* = 20). (**e–j**) The following parameters were determined in serum samples collected from human participants with T2D: HbA_1c_ and FBG (mmol/l). Total body mass, fat mass and lean mass were also analysed in human participants. The level of miR-193b was positively correlated with FBG (**e**) and fat mass/body weight (%) (**i**), while it negatively correlated with lean mass/body weight (%) (**j**) (*n* = 20). In (**b**–**j**): ***p < 0.001 vs control (normal control [NC] or C57) by unpaired Student’s *t* test

miR-sequencing (miR-seq) was performed on the TA of *db/db* mice and C57 mice. In Fig. 1a, miRs are sorted based on fold change, in descending order from top to bottom. miR-193b was significantly increased in *db/db* mice vs C57 mice (*p* < 0.05). miR-193b increased in the serum and skeletal muscle of *db/db* mice when compared with C57 mice, and similar results were observed in C57 mice fed an HFD vs a control diet (Fig. 1b, c and ESM Fig. 1a, b). Several clinical studies have suggested that the serum level of miR-193b is significantly increased in individuals with impaired glucose tolerance and type 2 diabetes [25, 26]. Our results also showed that serum miR-193b levels were significantly increased in individuals with type 2 diabetes vs healthy individuals (Fig. 1d). Next, we performed an analysis of miR-193b expression in healthy individuals and in those with type 2 diabetes. The circulating levels of miR-193b positively correlated with FBG (Fig. 1e) but not with HbA_1c_ (Fig. 1f), fat mass (Fig. 1g), lean mass (Fig. 1h), triacylglycerol or C-peptide (ESM Fig. 1c, d). Moreover, the circulating levels of miR-193b positively correlated with the ratio of fat mass/body weight (Fig. 1i) and negatively correlated with the ratio of muscle mass:body weight (Fig. 1j).

### Skeletal muscle miR-193b overexpression impaired muscle growth in wild-type mice

**Fig. 2:**
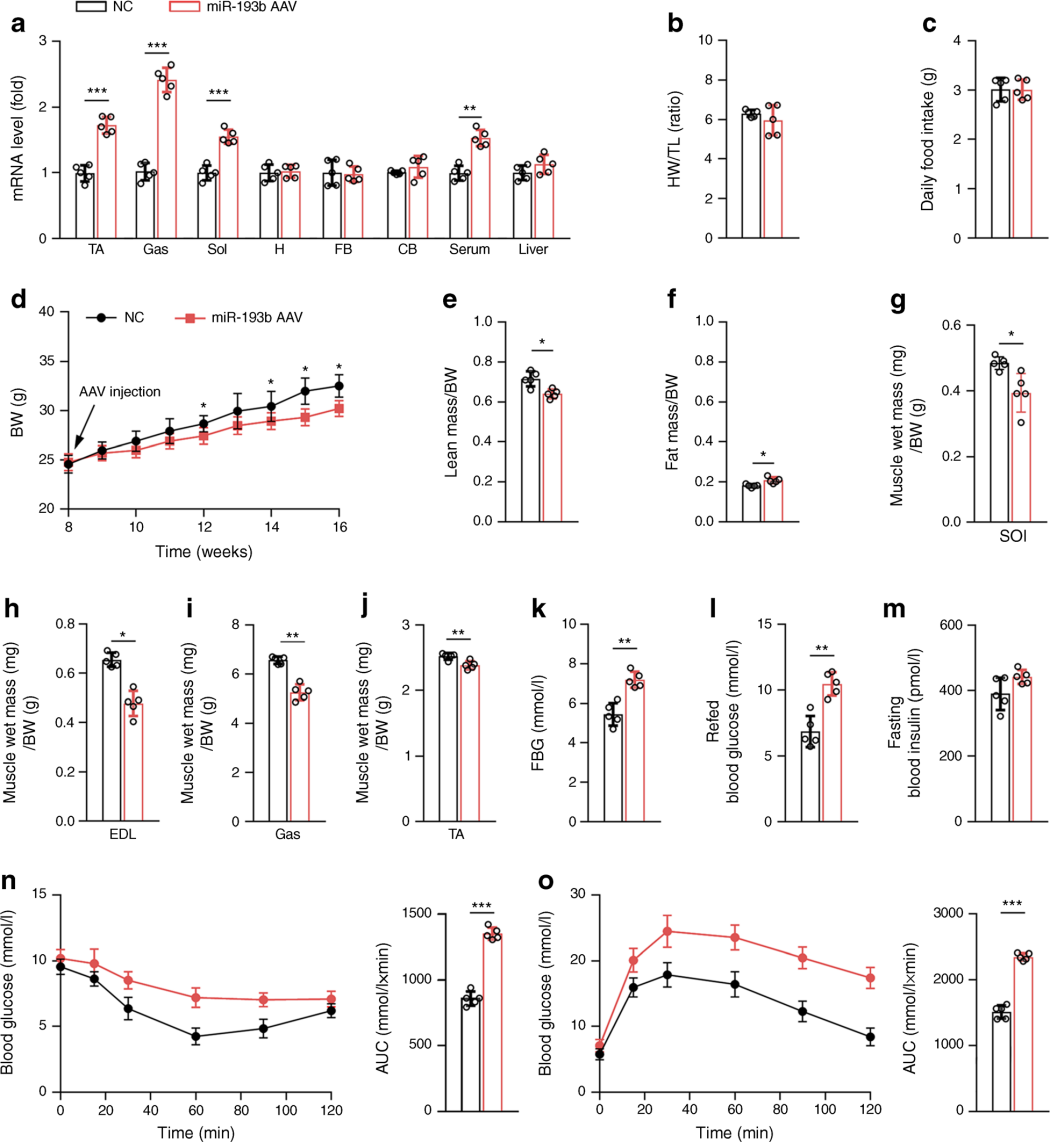
Skeletal muscle miR-193b overexpression impairs muscle mass and insulin sensitivity in C57 mice. C57 mice were injected with AAV-hsa-*gfp* (normal control [NC]) or miR-193b AAV through the tail vein at 8 weeks of age. (**a**) qPCR was used to determine miR-193b levels in the following samples: TA, gastrocnemius (Gas) muscle, soleus (Sol) muscle, heart (H), forebrain (FB), cerebellum (CB), serum and liver (*n* = 5). (**b**) The ratio of heart weight (HW; mg) and tibia length (TL; mm) (*n* = 5). (**c**) Daily food intake (*n* = 5). (**d**) Body weight (BW) changes over time (*n* = 5). *p < 0.05 vs NC at the same time point, by unpaired Student’s *t* test. (**e**, **f**) Body mass measured by MRI in 16-week-old mice (*n* = 5). (**g**–**j**) Weights of four muscle types, Sol (**g**), extensor digitorum longus (EDL; **h**), Gas (**i**) and TA (**j**), from NC mice or mice injected with miR-193b AAV for 8 weeks (*n* = 5). (**k**–**m**) FBG (**k**), refed blood glucose (**l**) and fasting blood insulin (**m**) (*n* = 5). (**n**, **o**) Blood glucose during ITT (**n**) and GTT (**o**) (*n* = 5). Key in (**a**) also applies to (**b**, **c**, **e–o**); key in (**d**) also applies to line graphs in (**n**, **o**). In (**a**) and (**e**–**o**), *p < 0.05, **p < 0.01, ***p < 0.001 vs NC, by unpaired Student’s *t* test

Systemic injection of AAV9 in vivo can efficiently target skeletal muscles and peripheral organs [27]. Muscle-specific *Acta1* (encoding hsa) promoter was used to drive target gene expression in muscle. First, AAV9-hsa-miR-193b was injected in situ in the TA of C57 mice and the results showed that miR-193b overexpression impaired muscle growth compared with controls (ESM Fig. 2a–c). Next, C57 mice were injected with miR-193b AAV via the tail vein and muscle was harvested 8 weeks after injection. Analysis of levels of miR-193b in various tissues verified the successful AAV-induced miR-193b overexpression in the skeletal muscles of mice (Fig. 2a), without influencing the heart weight/tibia length ratio and daily food intake, as compared with controls (Fig. 2b, c). We also found that miR-193b overexpression slightly but significantly reduced the body weight of mice vs controls (Fig. 2d). Moreover, further analysis of the body composition of mice showed that, compared with control mice, miR-193b AAV-infected mice exhibited significant muscle loss, while the ratio of fat content to body weight significantly increased (Fig. 2e, f). Consistent with this, the muscle mass of the soleus, extensor digitorum longus (EDL), gastrocnemius and TA muscles decreased after miR-193b AAV infection vs controls (Fig. 2g–j). Compared with the control mice, we found that skeletal muscle overexpression of miR-193b could induce insulin resistance in mice, as shown by increased fasting and refed glucose levels (Fig. 2k, l) in serum. Hence, glucose was elevated in the ITTs and GTTs (Fig. 2n, o), resulting in an increase in the AUC of glucose. Overall, miR-193b overexpression impaired muscle growth in miR-193b AAV-infected C57 mice vs controls.

### Skeletal muscle miR-193b overexpression reduced the performance and endurance of skeletal muscles

**Fig. 3:**
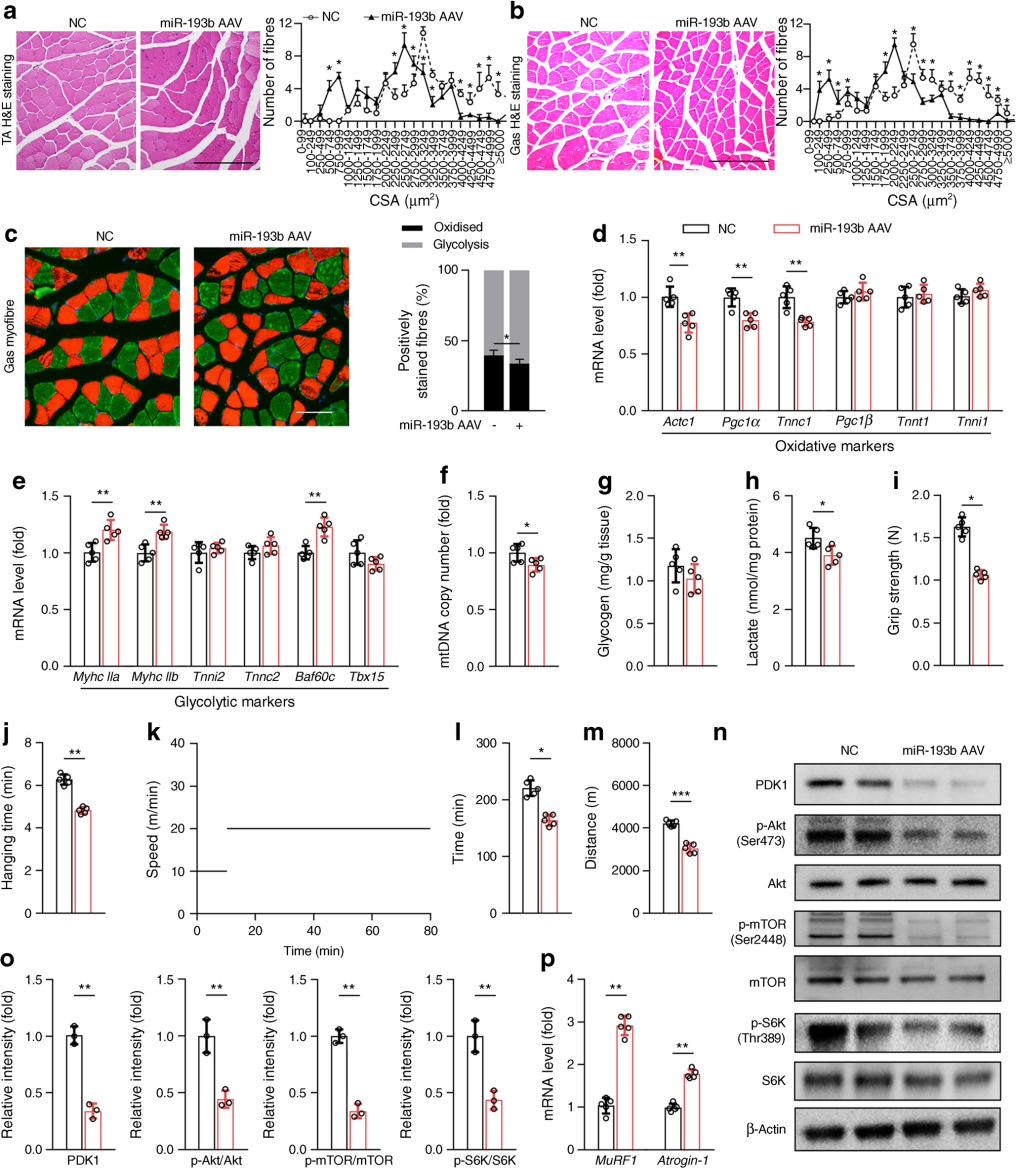
Skeletal muscle miR-193b overexpression impairs skeletal muscle function and inhibits the PDK1/Akt/mTOR signalling pathway. (**a**, **b**) Histology of TA (**a**) and gastrocnemius (Gas; **b**) muscles, as determined by H&E staining in both the normal control (NC) group and miR-193b AAV-treated group (scale bars, 250 μm), and quantitative analysis of findings (*n* = 5). *p < 0.05, miR-193b AAV-treated vs NC mice at the same CSA size range, by unpaired Student’s *t* test. (**c**) Immunofluorescent staining for myofibre type in Gas muscles (oxidative fibres, green; glycolytic fibres, red; scale bar, 100 μm) and quantitative analysis of findings (*n* = 3). (**d**, **e**) qPCR analysis was used to detect mRNA levels of *Actc1*, *Pgc1α* (also known as *Ppargc1α*), *Tnnc1*, *Pgc1β* (also known as *Ppargc1β*), *Tnnt1* and *Tnni1* (**d**), and *Myhc IIa* (also known as *Myh2*), *Myhc IIb* (also known as *Myh4*), *Tnni2*, *Tnnc2*, *Baf60c* (also known as *Smarcd3*) and *Tbx15* (**e**) in Gas muscle (*n* = 5) from AAV-hsa-*gfp-*treated mice (NC, black bars) and miR-193b AAV-treated mice (red bars). (**f**–**h**) mtDNA copy number (**f**), and glycogen (**g**) and lactate (**h**) content in Gas muscle were analysed using assay kits according to the manufacturers’ instructions (*n* = 5). (**i**–**k**) Forelimb grip strength (**i**), maximum hanging time (**j**), the pattern of treadmill speed for training to exhaustion (**k**), running time (**l**) and distance to exhaustion (**m**) for both mouse groups at 16 weeks of age (*n* = 5). (**n**, **o**) Western blot analysis of the expression of PDK1, p-Akt, Akt, p-mTOR, mTOR, p-S6K and S6K in Gas muscles from C57 mice injected with AAV-hsa-*gfp* (NC) or AAV-miR-193b for 8 weeks (**n**). Quantification of the relative levels of PDK1, p-Akt/Akt, p-mTOR/mTOR and p-S6K/S6K proteins is also shown (**o**; *n* = 3). (**p**) qPCR analysis of the mRNA level of *MuRF1* and *Atrogin*-1 (*n* = 5). Key in (**a**) also applies to (**b**); key in (**d**) also applies to (**e**–**j**, **l**, **m**, **o**, **p**). In (**c**–**f**) and (**h**–**j**, **l**, **m**, **o**, **p**), *p < 0.05, **p < 0.01, ***p < 0.001, by unpaired Student’s *t* test

Basic histological evaluation of the glycolytic TA and gastrocnemius muscles revealed that the miR-193b AAV-infected mice had a normal muscle structure as compared with control mice (Fig. 3a, b). In contrast, the TA and gastrocnemius of miR-193b AAV-infected mice had a larger number of small myofibres and exhibited a distribution profile that shifted to the left (Fig. 3a, b). Additionally, an examination of the myofibre size and types revealed that miR-193b treatment slightly decreased the ratio of oxidative/glycolytic myofibres, according to immunofluorescent staining results (Fig. 3c). mRNA levels of myofibre oxidative and glycolytic markers also support our findings (Fig. 3d, e). Compared with glycolytic muscle fibres, oxidative muscle fibres have more mitochondria and less muscle glycogen and lactic acid [28]. Skeletal muscle miR-193b overexpression was slightly elevated in the glycolytic muscle fibres, as shown by decreased mtDNA copy number and lactate content in miR-193b-treated mice vs controls, but there was no difference in glycogen levels between the groups (Fig. 3f–h). Furthermore, our results indicated that miR-193b overexpression weakened the grip strength of the forelimb of mice and reduced hanging time, exhaustive running time and maximum running distance vs controls (Fig. 3i–m). Thus, miR-193b overexpression was shown to reduce muscle performance and endurance by decreasing the cross-sectional area (CSA) of myofibres and by increasing the ratio of glycolytic muscle to oxidative muscle to a small extent without influencing normal muscle structure.

The CSA of myofibres is controlled by the balance between protein synthesis and degradation [15]. Activated Akt activates mTOR/S6K signalling pathway-mediated protein synthesis and inhibits FOXO1-mediated protein degradation to increase muscle protein content [10]. Compared with controls, miR-193b overexpression reduced PDK1 expression and phosphorylation of Akt, mTOR, S6K and AMPK (Fig. 3n, o and ESM Fig. 3a), while it enhanced the expression of *Atrogin-1* and *MuRF1* (muscle-specific ubiquitin ligases; Fig. 3p), which are both downstream targets of FOXO1 [12].

Collectively, these data suggest that miR-193b overexpression leads to muscle loss and weakness, as well as attenuated PDK1/AKT/mTOR/S6K activation in muscles.

### Skeletal muscle miR-193b deficiency increased muscle mass in C57 mice

C57 mice were injected with sgmiR-193b AAV via the vein tail and the muscle was harvested 8 weeks after injection. Compared with untreated mice, sgmiR-193b AAV injection significantly increased the body weight and muscle mass of C57 mice (ESM Fig. 4d, e). The evaluation of the soleus, EDL, gastrocnemius and TA muscles further confirmed our findings, in that the mass of these muscles increased after sgmiR-193b AAV infection (ESM Fig. 4g). Mice had normal muscle structure but fewer small myofibres after sgmiR-193b AAV injection in the TA and gastrocnemius muscles, as compared with controls (ESM Fig. 4h, i). Thus, skeletal muscle miR-193b deficiency was shown to increase muscle mass in C57 mice.

### Skeletal muscle miR-193b deficiency improved muscle loss in *db/db* mice

**Fig. 4:**
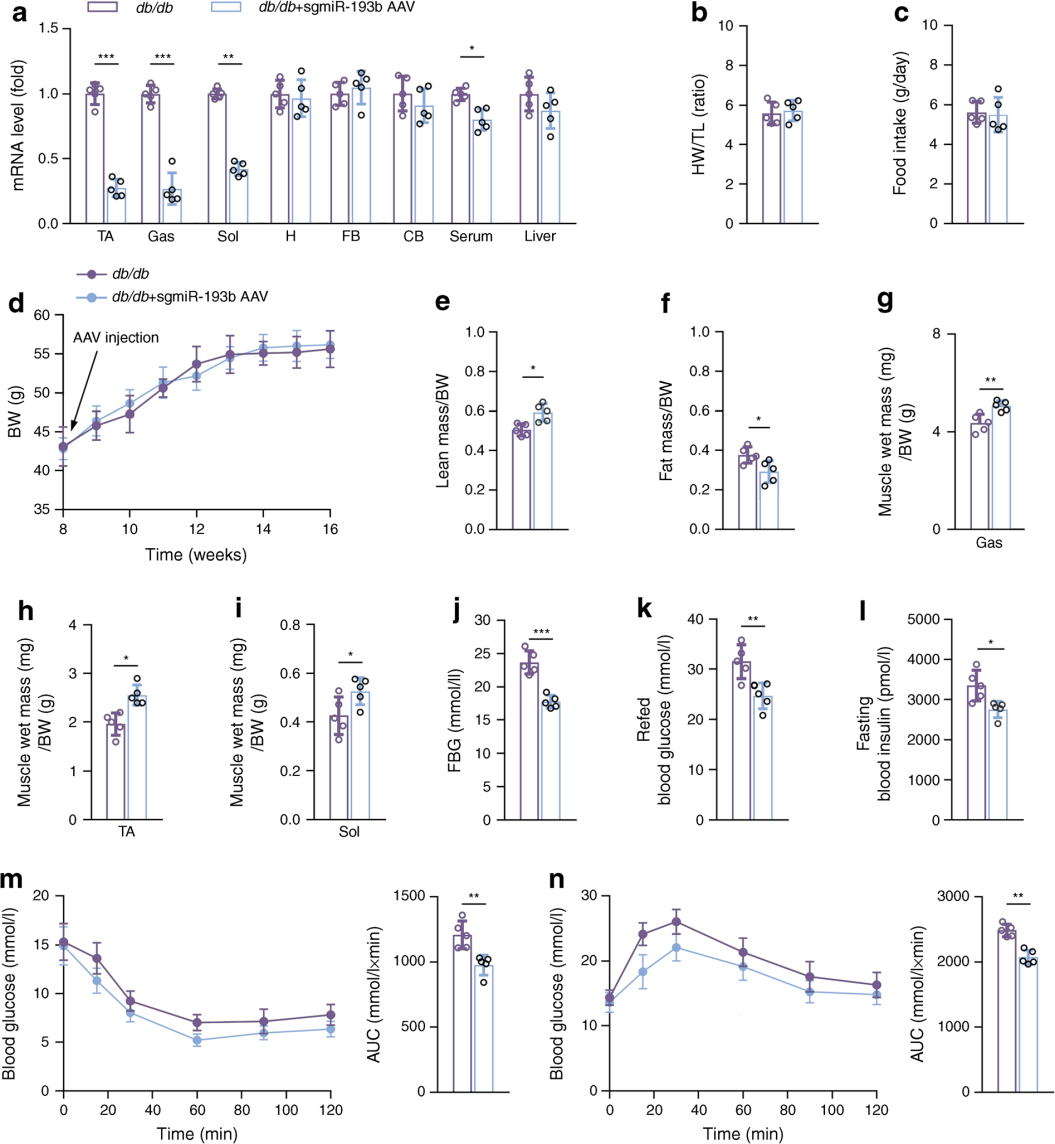
Skeletal muscle miR-193b-deficiency increases muscles mass and insulin sensitivity in mouse models of diabetes. *db/db* mice were injected with AAV-hsa-*gfp* (control mice, labelled ‘*db*/*db*’) or AAV-hsa-sgmiR-193b through the tail vein at 8 weeks of age. (**a**) qPCR was used to determine miR-193b levels in the following samples: TA, gastrocnemius (Gas) muscle, soleus (Sol) muscle, heart (H), forebrain (FB), cerebellum (CB), serum and liver (*n* = 5). (**b**) The ratio of heart weight (HW; mg) and tibia length (TL; mm) (*n* = 5). (**c**) Daily food intake (*n* = 5). (**d**) Body weight (BW) changes over time (*n* = 5). (**e**, **f**) Body mass measured by MRI at 16 weeks of age (*n* = 5). (**g**–**i**) Weights of three muscle types, Gas (**g**), TA (**h**) and Sol (**i**), from *db/db* mice or mice injected with AAV-hsa-sgmiR-193binjected for 8 weeks (*n* = 5). (**j**–**l**) FBG (**j**), refed blood glucose (**k**) and fasting blood insulin (**l**) (*n* = 5). (**m**, **n**) Blood glucose during ITT (**m**) and GTT (**n**) (*n* = 5). Key in (**a**) also applies to (**b**, **c**, **e**–**n**); key in (**d**) also applies to line graphs in (**m**, **n**). *p < 0.05, **p < 0.01, ***p < 0.001 vs *db/db*, by unpaired Student’s *t* test

Next, sgmiR-193b AAV was injected via the vein tail into *db/db* mice to create skeletal muscle miR-193b-deficient *db/db* mice. miR-193b expression was significantly inhibited in various muscles of the mice injected with sgmiR-193b AAV vs untreated *db*/*db* mice (Fig. 4a), without influencing heart weight/tibia length ratio or daily food intake (Fig. 4b, c). The body weight of *db/db* mice did not significantly change after sgmiR-193b AAV injection vs controls (Fig. 4d); however, muscle mass was significantly increased and fat mass decreased (Fig. 4e, f). Skeletal muscle miR-193b deficiency enhanced locomotor activity in *db/db* mice, but not in C57 mice vs wild-type mice of the same genotype (ESM Fig. 5). The change in locomotor activity in *db/db* mice was only observed in the night and not in the day (ESM Fig. 5). Evaluation of the soleus, gastrocnemius and TA further confirmed that muscle mass increased after sgmiR-193b AAV infection vs untreated controls (Fig. 4g–i). Additionally, skeletal muscle miR-193b deficiency enhanced insulin sensitivity in *db/db* mice vs controls (Fig. 4j–n). Thus, skeletal muscle miR-193b deficiency was shown to attenuate muscle loss in *db/db* mice.

### Skeletal muscle miR-193b deficiency improved skeletal muscle performance and endurance in *db/db* mice

**Fig. 5:**
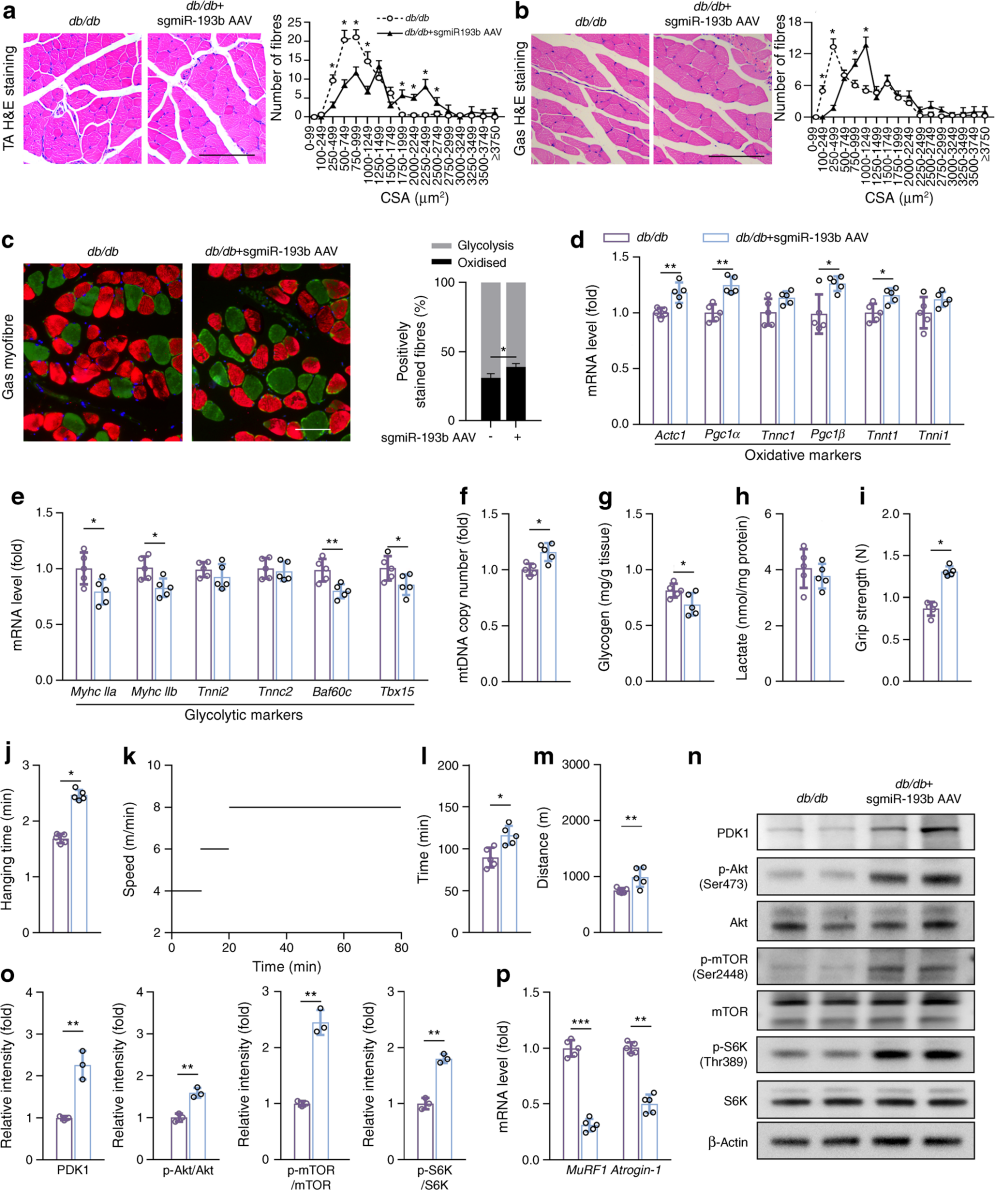
miR-193b-deficiency improves skeletal muscle function and activates the PDK1/Akt/mTOR signalling pathway. (**a**, **b**) Histology of TA (**a**) and gastrocnemius (Gas; **b**) muscles as determined by H&E staining in *db/db* mice injected with AAV-hsa-*gfp* (‘*db/db*’ group) and *db/db* mice injected with sgmiR-193b AAV (‘*db/db* + sgmiR-193b AAV’ group) (scale bars, 150 μm), and quantitative analysis of findings (*n* = 5). *p < 0.05, *db*/*db* + sgmiR-193b AAV group vs *db*/*db* group at the same CSA size range, by unpaired Student’s *t* test. (**c**) Immunofluorescent staining for myofibre type in Gas muscles (oxidative fibres, green; glycolytic fibres, red; scale bar, 100 μm) and quantitative analysis of findings (*n* = 3). (**d**, **e**) qPCR analysis was used to detect mRNA level of *Actc1*, *Pgc1α*, *Tnnc1*, *Pgc1β*, *Tnnt1* and *Tnni1* (**d**), and *Myhc IIa*, *Myhc IIb*, *Tnni2*, *Tnnc2*, *Baf60c* and *Tbx15* (**e**) in Gas muscle (*n* = 5). (**f**–**h**) mtDNA copy number (**f**), and glycogen (**g**) and lactate (**h**) content in Gas muscle were analysed using assay kits according to the manufacturers’ instructions (*n* = 5). (**i**–**k**) Forelimb grip strength (**i**), maximum hanging time (**j**), the pattern of treadmill speed for training to exhaustion (**k**), running time (**l**) and distance to exhaustion (**m**) for both mouse groups at 16 weeks of age (*n* = 5). (**n**, **o**) Western blot analysis of the expression of PDK1, p-Akt, Akt, p-mTOR, mTOR, p-S6K and S6K in Gas muscles from *db/db* mice injected with AAV-hsa-*gfp* or AAV-hsa-sgmiR-193b for 8 weeks (**n**). Quantification of the relative levels of PDK1, p-Akt, p-mTOR and p-S6K proteins is also shown (**o**; *n* = 3). (**p**) qPCR analysis was used to determine the mRNA level of *MuRF1* and *Atrogin-1* (*n* = 5). Key in (**a**) also applies to (**b**); key in (**d**) also applies to (**e**–**j**, **l**, **m**, **o**, **p**). In (**c**–**m**) and (**l**, **m o**, **p**), *p < 0.05, **p < 0.01, ***p < 0.001, by unpaired Student’s *t* test

According to our results, mouse models of diabetes had normal muscle structure but fewer small myofibers after sgmiR-193b AAV injection in the TA and gastrocnemius muscles, as compared with untreated controls (Fig. 5a, b). Immunofluorescent staining showed that miR-193b deficiency gently enhanced the ratio of oxidative myofibres/glycolytic myofibres (Fig. 5c). The mRNA levels of oxidative markers and glycolytic markers, mtDNA copy numbers and glycogen content also supported these findings, but there was no difference in lactate levels between the groups (Fig. 5d–h). Furthermore, sgmiR-193b AAV injection significantly enhanced the hanging time, grip strength, and maximum running distance and time in *db/db* mice (Fig. 5i–m). Additionally, the phosphorylation of Akt, mTOR, S6K and AMPK and the expression of PDK1 were significantly increased (Fig. 5n, o and ESM Fig. 3b), while the expression levels of *Atrogin-1* and *MuRF1* were decreased (Fig. 5p) after sgmiR-193b AAV injection in diabetic mice vs vehicle-treated controls. Thus, we demonstrated that miR-193b deficiency attenuated muscle loss and weakness in *db/db* mice, as well as activating the PDK1/AKT/mTOR/S6K signalling pathway.

### miR-193b reduced the number and size of myotubes formed in C2C12 cells

Compared with control cells, the miR-193b mimic was found to significantly inhibit myotube formation in C2C12 cells, in terms of both number and size of myotubes (ESM Fig. 6a). Likewise, treatment with the miR-193b mimic reduced Myh expression in C2C12 cells vs control cells (ESM Fig. 6b). In contrast, miR-193b inhibitor treatment enhanced myotube formation and increased Myh protein levels in C2C12 cells vs controls (ESM Fig. 6c, d). Treatment with DEX alone greatly reduced both the number and size of the myotubes vs untreated cells. Co-treatment of cells with DEX and the miR-193b inhibitor attenuated the DEX-induced reduction in myotube formation (ESM Fig. 6e–g). Finally, when compared with untreated cells, DEX or TNF-α treatment induced miR-193b expression in a time-dependent manner (ESM Fig. 6h). Overall, these results suggest that DEX or inflammation could induce miR-193b upregulation in the skeletal muscle and that miR-193b impairs muscle growth.

### miR-193b interferes with Akt activation by targeting PDK1

**Fig. 6:**
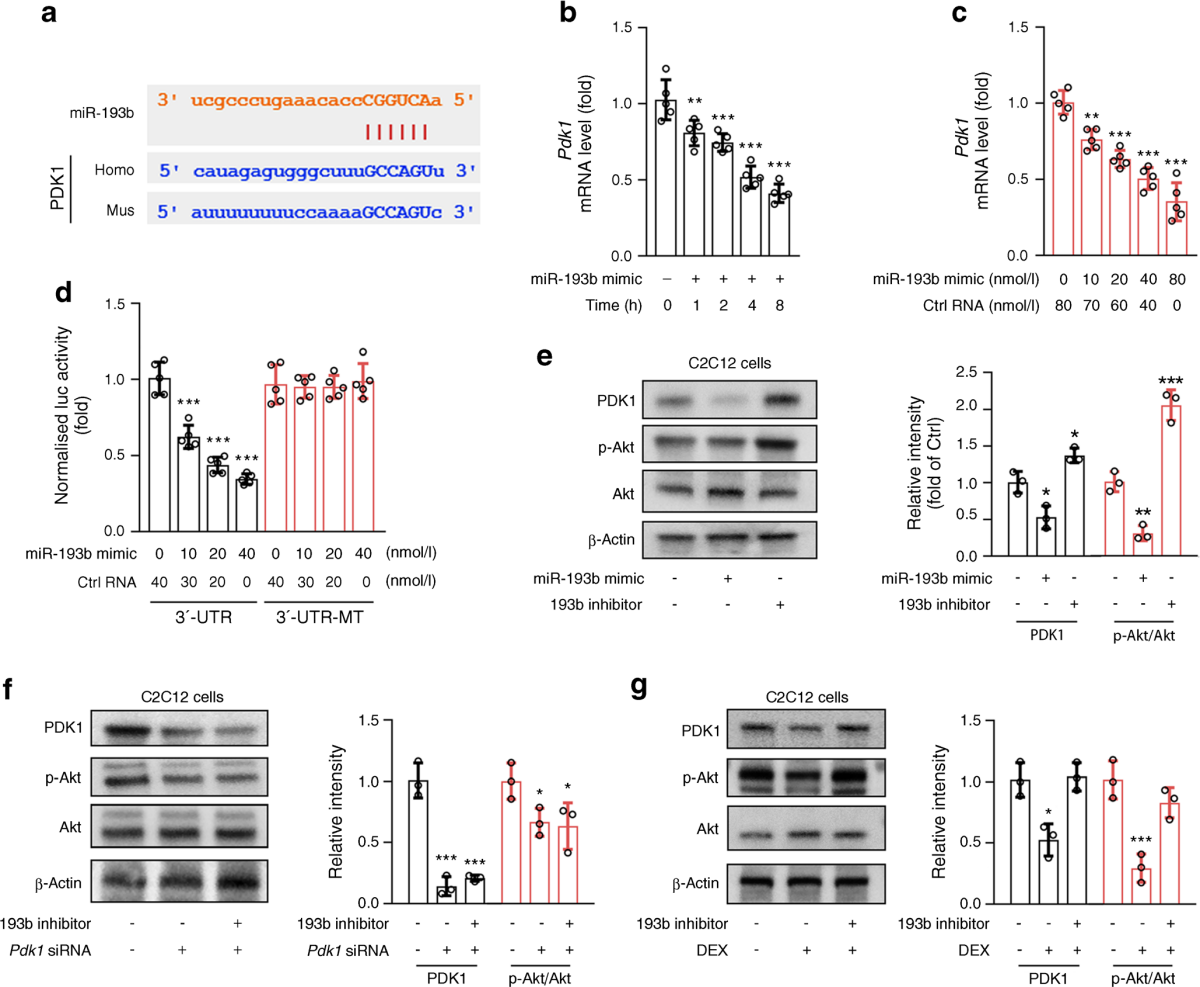
miR-193b decreases activation of Akt by inhibiting PDK1 expression. (**a**) Graphical representation of the conserved miR-193b binding motifs within the 3′-UTR of *Pdk1*. Complementary sequences to the seed regions of miR-193b within the 3′-UTRs are conserved between human (Homo) and mouse (Mus) sequences. (**b**, **c**) C2C12 cells were treated with miR-193b mimic or control (Ctrl) RNA and qPCR analysis was used to determine the mRNA level of PDK1 (*n* = 5). **p < 0.01, ***p < 0.001 vs time 0 (**b**) or Ctrl RNA treatment alone (**c**), by one-way ANOVA with Bonferroni correction. (**d**) Luciferase (luc) activity of the reporter constructs containing either wild-type or mutated (MT) 3′-UTR of murine *Pdk1* after treatment of C2C12 cells with miR-193b mimic or Ctrl RNA (*n* = 5). ***p < 0.001 vs Ctrl RNA treatment alone in the 3′-UTR-transfected group, by one-way ANOVA with Bonferroni correction. (**e**) C2C12 cells were treated with miR-193b mimic (40 nmol/l) or miR-193b inhibitor (100 nmol/l) and the protein level of PDK1, Akt and p-Akt was detected by western blot analysis. Quantification of the relative levels of PDK1 and p-Akt proteins is shown (*n* = 3). (**f**) *Pdk1* siRNA was transfected into C2C12 cells. Cells were then treated with miR-193b inhibitor or nontargeting negative Ctrl RNA and western blot analysis was used to determine the protein level of PDK1, Akt and p-Akt. Quantification of the relative levels of PDK1 and p-Akt proteins is shown (*n* = 3). (**g**) C2C12 cells were treated with DEX alone or with a combination of miR-193b inhibitor and DEX and the protein level of PDK1, Akt and p-Akt was detected by western blot. Quantification of the relative levels of PDK1 and p-Akt proteins is shown (*n* = 3). In (**e**–**g**): *p < 0.05, **p < 0.01, ***p < 0.001 vs untreated cells, by one-way ANOVA with Bonferroni correction

Research has shown that miR-193b deficiency leads to increased basal and cytokine-induced Akt signalling in haematopoietic stem and progenitor cells [29]. PDK1 is a master kinase that phosphorylates and activates a subgroup of the AGC family of protein kinases (including Akt) [30]. Using StarBase (http://starbase.sysu.edu.cn/, accessed 5 January 2020) [31], we identified *PDK1* as a potential target of miR-193b (Fig. 6a). When compared with control RNA treatment, the miR-193b mimic reduced the mRNA level of *Pdk1* in a time- and dose-dependent manner in C2C12 cells (Fig. 6b, c). Next, a dual luciferase reporter assay was used to show that the activity of luciferase reporters was markedly reduced with miR-193b mimic treatment in a dose-dependent manner vs treatment with control RNA alone, while reporter activity was not affected following treatment with the *Pdk1* 3′- UTR—mutant luciferase reporter construct (Fig. 6d), suggesting that *Pdk1* is the direct target gene of miR-193b. Analysis of protein levels in C2C12 cells by western blot assay also supported our findings that miR-193b inhibits the expression of PDK1 and phosphorylation (activation) of Akt, as compared with untreated controls (Fig. 6e). Notably, PDK1 deficiency impaired the regulation of Akt activation by the miR-193b inhibitor in C2C12 cells (Fig. 6f). Furthermore, DEX treatment of C2C12 cells resulted in decreased Akt activation vs untreated cells (Fig. 6g); however, co-treatment of cells with DEX and the miR-193b inhibitor reversed the DEX-induced inactivation of Akt. Thus, miR-193b was demonstrated to regulate Akt activation by inhibiting PDK1 expression by directly binding to the *Pdk1* 3′-UTR.

### PDK1 is required for miR-193b-mediated inhibition of muscle growth in wild-type mice

**Fig. 7:**
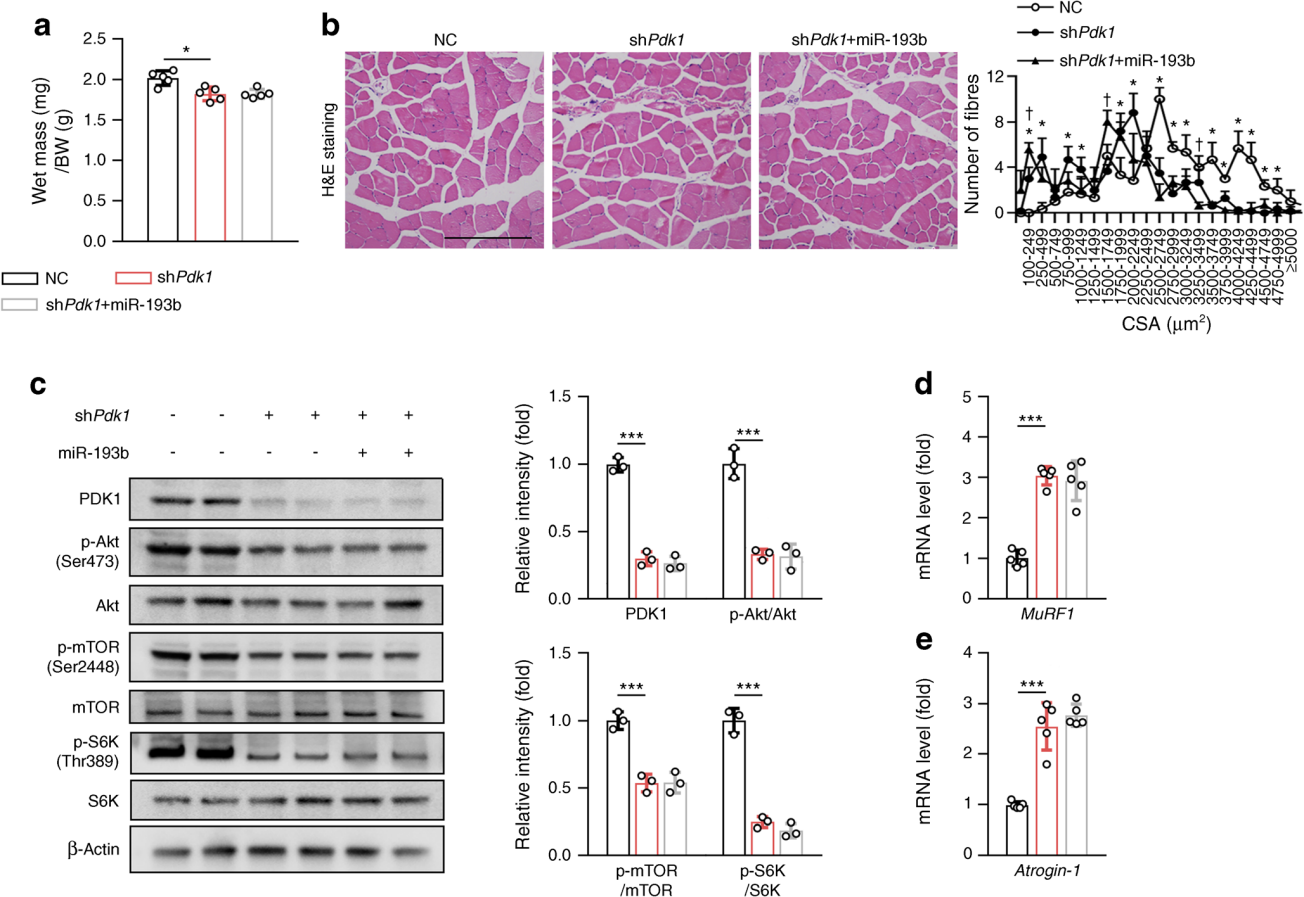
PDK1-deficiency abrogates miR-193b-mediated muscle atrophy. (**a**) Weights of TA muscle in normal control (NC), sh*Pdk1* AAV-infected and sh*Pdk1* AAV + miR-193b AAV-infected C57 mice (*n* = 5). BW, body weight. (**b**) Histology of TA muscles as determined by H&E staining in the NC, sh*Pdk1* AAV and sh*Pdk1* AAV + miR-193b AAV mouse groups (scale bar, 250 μm), and quantitative analysis of findings (*n* = 5). *p < 0.05, sh*Pdk1* vs NC at the same CSA size range; ^†^*p* < 0.05, sh*Pdk1* + miR-193b vs sh*Pdk1* at the same CSA size range; analysed by one-way ANOVA with Bonferroni correction. (**c**) Western blot analysis of the expression of PDK1, p-Akt, Akt, p-mTOR, mTOR, p-S6K and S6K in TA muscles from NC, sh*Pdk1* AAV and sh*Pdk1* AAV + miR-193b AAV mouse groups. Quantification of the relative levels of PDK1, p-Akt, p-mTOR and p-S6K proteins is also shown (*n* = 3). (**d**, **e**) mRNA levels of *MuRF1* (**d**) and *Atrogin-1* (**e**), as determined by qPCR (*n* = 5). Key in (**a**) also applies to (**c**–**e**). In (**a**, **c**–**e**): *p < 0.05, ***p < 0.001 vs NC, by one-way ANOVA with Bonferroni correction

sh*Pdk1* AAV-infected TA muscles exhibited a mild but significant muscle loss that was unchanged by miR-193b AAV co-treatment when compared with vehicle-treated muscles (Fig. 7a). H&E staining of the TA showed that PDK1-deficient mice had a large number of small myofibers compared with control tissue (Fig. 7b). Moreover, in contrast to the previous observation that miR-193b AAV treatment resulted in a larger number of small myofibres (Fig. 3b), the CSA profile of sh*Pdk1* AAV-only treated mice was apparently similar to the profile of mice treated with sh*Pdk1* AAV + miR-193b AAV, indicating that PDK1 deficiency blocked the effect of miR-193b on myofibre size. Consistent with this finding, as compared with control tissue, Akt, mTOR and S6K activation were greatly decreased by PDK1 depletion, and these effects were unaltered by co-treatment of tissue with sh*Pdk1* and miR-193b AAVs (Fig. 7c). Additionally, as compared with control tissue, the expression of protein degradation genes (*MuRF1* and *Atrogin-1*) in mouse TA was significantly increased in PDK1-deficient mice and this finding was unaffected by co-treatment with sh*Pdk1* and miR-193b AAVs (Fig. 7d, e). Moreover, compared with untreated mice, the activation of AMPK in mouse TA was reduced after sh*Pdk1* AAV infection in C57 mice and this finding did not change with co-treatment of mice with sh*Pdk1* and miR-193b AAVs (ESM Fig. 3c). Overall, these data suggest that miR-193b impairs muscle growth by regulating the Akt/mTOR/S6K pathway by inhibiting PDK1.

### Skeletal muscle insulin resistance may upregulate the miR-193b expression through the mTOR/DNA methyltransferase signalling pathway

Methylation of the miR-193b promoter [32–34], which could lead to inhibition of miR-193b expression, has been reported in several tumours [32–34]. Interestingly, our analysis of the GEO database entry GSE29221 revealed that DNA methyltransferase (DNMT1) expression was significantly decreased in the skeletal muscles of individuals with type 2 diabetes (*n* = 11) as compared with those without type 2 diabetes (*n* = 13) (153.1 ± 19.2 vs 283.9 ± 181.2; *p* = 0.013). When compared with untreated cells, we also found that DEX or TNF-α treatment of C2C12 cells induced insulin resistance, as demonstrated by the inactivation of mTOR (ESM Fig. 7a, b), and reduced *Dnmt1* expression (ESM Fig. 7c, e) and enhanced expression of miR-193b (ESM Fig. 7d, f). This evidence suggests that chronic inflammation- or DEX-induced insulin resistance could enhance miR-193b expression in the C2C12 cells.

## Discussion

In this study, we described the effect of miR-193b, a predicted inhibitor of PDK1, using bioinformatics (starbase.sysu.edu.cn [31]). In line with this prediction, miR-193b decreased PDK1 expression, which was accompanied by a decrease in Akt activity and Akt-regulated protein synthesis. These findings are consistent with those of previous studies that have demonstrated that miR-193b deficiency in various tumour cells leads to increased Akt activation [29, 35, 36]*.* Remarkably, we found that miR-193b deficiency attenuated muscle loss and enhanced muscle function in *db/db* mice.

Interestingly, skeletal muscle miR-193b deficiency in C57 mice resulted in increased body weight compared with wild-type mice (ESM Fig. 4d). In contrast, the body weight of *db/db* mice did not significantly change after sgmiR-193b AAV injection vs control mice (Fig. 4d). Following these findings, we conducted an analysis of locomotor activity in mice using metabolic cages and found that skeletal muscle miR-193b-deficiency significantly enhanced night-time locomotor activity in *db/db* mice compared with controls (ESM Fig. 5a). It is well known that locomotor activity or voluntary wheel running activity of *db/db* mice is significantly lower than that of wild-type mice [37, 38]. Furthermore, our results showed that sgmiR-193b AAV treatment did not significantly influence the locomotor activity of C57 mice (ESM Fig. 5b). In addition, compared with controls, miR-193b expression in the cerebellum and forebrain of C57 and *db*/*db* mice was not affected by miR-193b AAV or sgmiR-193b AAV injection (Figs. 2a and 4a). Taken together, we speculate that loss of miR-193b may improve fatigue in *db/db* mice, resulting in increased locomotor activity and reduced fat mass (Fig. 4f). However, more experiments are needed to test this hypothesis.

miR-193b, a well-known tumour suppressor, is widely expressed in the body, especially in the muscles [39]. Recent studies on miR-193b function have mainly concentrated on tumour viability and migration. They have also shown that miR-193b controls adiponectin production in human white adipose tissue [40]. Additionally, miR-193b overexpression downregulated C-C motif chemokine ligand 2 (CCL2) production indirectly through a network of transcription factors in a human monocyte/macrophage cell line [41]. Another study showed that miR-193a/b-3p overexpression attenuates liver fibrosis by suppressing the proliferation and activation of hepatic stellate cells (HSCs) via repression of collagen type I α1 (Col1α1) and α-smooth muscle actin expression, and by inhibiting the activation of the TGF-β/Smad signalling pathway [42]. However, the role of miR-193b in modulating skeletal muscle function has rarely been reported. Regarding C2C12 cells, our data suggest that miR-193b inhibits the Akt/mTOR/S6K axis by reducing PDK1 expression. Furthermore, inactivation of the Akt/mTOR/S6K axis by miR-193b appears to be a key player in the development of muscle weakness and loss in *db/db* mice.

Upon initiation of resistance exercise, there is an immediate increase in ‘anabolic’ kinase activity, including activation of the Akt/S6K pathway. Following energy consumption, redox potentials activate ‘metabolic’ kinases, such as AMPK, which ultimately enhance muscle function through the regulation of mitochondrial protein synthesis and oxidative capacity [43]. Consistent with our results, miR-193b deficiency enhanced the expression of PDK1 in *db/db* mice, followed by activation of the Akt/mTOR/S6K pathway, as well as the AMPK signalling pathway, and decreased the expression of *MuRF1* and *Atrogin-1* (Fig. 5l, m and ESM Fig. 3b). Furthermore, H&E staining showed that miR-193b deficiency improved skeletal muscle loss in *db/db* mice (Fig. 5a, b). AMPK is a master regulator of mitochondrial mass [44]. We found that miR-193b deficiency mildly increased oxidative muscle fibres in the skeletal muscle of *db/db* mice, as compared with controls, and increased mitochondrial mass in skeletal muscle (Fig. 5c–h), which was perhaps caused by the activation of AMPK in the skeletal muscles of *db/db* mice (ESM Fig. 3b). Therefore, inhibition of miR-193b could enhance muscular hypertrophy, strength, power and local muscular endurance in *db/db* mice, in a way that represents muscle adaptation responses to resistance exercise. Thus, inhibition of miR-193b may have great potential in mimicking resistance exercise.

The insights obtained from this study contribute to our understanding of the physiological roles of miR-193b in regulating the PDK1/Akt signalling pathway and muscle loss, which is important for addressing the complexity of the development of type 2 diabetes-associated muscle atrophy. Notably, miR-193b and PDK1, as well as their expression patterns, are similar between humans and mice, suggesting that miR-193b could be a potential therapeutic target for type 2 diabetes-associated muscle atrophy in humans.

## Supplementary information

ESM (PDF 1539 kb)

## Data Availability

The data that support the findings of this study are available from the corresponding author (LJ) upon reasonable request.

## Abbreviations

AAV: Adeno-associated virus
AMPK: AMP-activated protein kinase
CSA: Cross-sectional area
DEX: Dexamethasone
EDL: Extensor digitorum longus
FBG: Fasting blood glucose
FOXO1: Forkhead box protein O1
GEO: Gene Expression Omnibus
HFD: High-fat diet
hsa: Human α-skeletal actin
miR: MicroRNA
mtDNA: Mitochondrial DNA
mTOR: Mammalian target of rapamycin
MuRF1: Muscle really interesting new gene-finger protein-1
Myh: Myosin heavy chain 7B
NIH: National Institutes of Health
PDK1: 3-Phosphoinositide-dependent protein kinase-1
PI3K: Phosphatidylinositol 3-kinase
qPCR: Quantitative real-time PCR
sgmiR-193b AAV: Adeno-associated virus-human α-skeletal actin-microRNA-193b-sponge
sh: Short hairpin
siRNA: Small interfering RNA
S6K: p70S6 kinase
TA: Tibialis anterior
UTR: Untranslated region
vg: Viral genomes

## References

[1] Chan J, Zhang Y, Ning G (2014). Diabetes in China: a societal solution for a personal challenge. Lancet Diabetes Endocrinol.

[2] Wang L, Gao P, Zhang M (2017). Prevalence and ethnic pattern of diabetes and prediabetes in China in 2013. JAMA.

[3] Ma R (2018). Epidemiology of diabetes and diabetic complications in China. Diabetologia.

[4] Kinney J (2004). Nutritional frailty, sarcopenia and falls in the elderly. Curr Opin Clin Nutr Metab Care.

[5] Cruz-Jentoft A, Baeyens J, Bauer J (2010). Sarcopenia: European consensus on definition and diagnosis: report of the European working group on sarcopenia in older people. Age Ageing.

[6] Trierweiler H, Kisielewicz G, Hoffmann Jonasson T, Rasmussen Petterle R, Aguiar Moreira C, Zeghbi Cochenski Borba V (2018). Sarcopenia: a chronic complication of type 2 diabetes mellitus. Diabetol Metab Syndr.

[7] Ogama N, Sakurai T, Kawashima S (2019). Association of Glucose Fluctuations with sarcopenia in older adults with type 2 diabetes mellitus. J Clin Med.

[8] Kim T, Park M, Yang S (2010). Prevalence and determinant factors of sarcopenia in patients with type 2 diabetes: the Korean Sarcopenic obesity study (KSOS). Diabetes Care.

[9] Czech M (2017). Insulin action and resistance in obesity and type 2 diabetes. Nat Med.

[10] Kim H, Cho S, Jeong H (2020). Indoprofen prevents muscle wasting in aged mice through activation of PDK1/AKT pathway. J Cachexia Sarcopenia Muscle.

[11] Schiaffino S, Mammucari C (2011). Regulation of skeletal muscle growth by the IGF1-Akt/PKB pathway: insights from genetic models. Skelet Muscle.

[12] Schiaffino S, Dyar K, Ciciliot S, Blaauw B, Sandri M (2013). Mechanisms regulating skeletal muscle growth and atrophy. FEBS J.

[13] Crossland H, Constantin-Teodosiu D, Gardiner S, Constantin D, Greenhaff P (2008). A potential role for Akt/FOXO signalling in both protein loss and the impairment of muscle carbohydrate oxidation during sepsis in rodent skeletal muscle. J Physiol.

[14] Zhang A, Li M, Wang B, Klein J, Price S, Wang X (2018). miRNA-23a/27a attenuates muscle atrophy and renal fibrosis through muscle-kidney crosstalk. J Cachexia Sarcopenia Muscle.

[15] Li Z, Cai B, Abdalla B (2019). LncIRS1 controls muscle atrophy via sponging miR-15 family to activate IGF1-PI3K/AKT pathway. J Cachexia Sarcopenia Muscle.

[16] Shin Y, Kwon E, Lee S (2020). A subset of microRNAs in the Dlk1-Dio3 cluster regulates age-associated muscle atrophy by targeting Atrogin-1. J Cachexia Sarcopenia Muscle.

[17] Huber W, Carey V, Gentleman R (2015). Orchestrating high-throughput genomic analysis with Bioconductor. Nat Methods.

[18] Zhang J, Qin J, Su Y (2017). miR-193b-3p possesses anti-tumor activity in ovarian carcinoma cells by targeting p21-activated kinase 3. Biomed Pharmacother.

[19] Lai N, Wu D, Liang T (2020). Systemic exosomal miR-193b-3p delivery attenuates neuroinflammation in early brain injury after subarachnoid hemorrhage in mice. J Neuroinflammation.

[20] Tinsley F, Taicher G, Heiman M (2004). Evaluation of a quantitative magnetic resonance method for mouse whole body composition analysis. Obes Res.

[21] Ma C, Xia R, Yang S (2020). Formononetin attenuates atherosclerosis via regulating interaction between KLF4 and SRA in apoE mice. Theranostics.

[22] Yang S, Chen Y, Duan Y (2019). Therapeutic potential of NaoXinTong capsule on the developed diabetic nephropathy in db/db mice. Biomed Pharmacother.

[23] Katagiri T, Yamaguchi A, Komaki M (1994). Bone morphogenetic protein-2 converts the differentiation pathway of C2C12 myoblasts into the osteoblast lineage. J Cell Biol.

[24] Chen Z, Yang J, Zhong J (2020). MicroRNA-193b-3p alleviates focal cerebral ischemia and reperfusion-induced injury in rats by inhibiting 5-lipoxygenase expression. Exp Neurol.

[25] Stępień E, Durak-Kozica M, Kamińska A (2018). Circulating ectosomes: determination of angiogenic microRNAs in type 2 diabetes. Theranostics.

[26] Párrizas M, Brugnara L, Esteban Y (2015). Circulating miR-192 and miR-193b are markers of prediabetes and are modulated by an exercise intervention. J Clin Endocrinol Metab.

[27] Bevan A, Duque S, Foust K (2011). Systemic gene delivery in large species for targeting spinal cord, brain, and peripheral tissues for pediatric disorders. Mol Ther.

[28] Wang Y, Zhang C, Yu R (2004). Regulation of muscle fiber type and running endurance by PPARdelta. PLoS Biol.

[29] Haetscher N, Feuermann Y, Wingert S (2015). STAT5-regulated microRNA-193b controls haematopoietic stem and progenitor cell expansion by modulating cytokine receptor signalling. Nat Commun.

[30] Lawlor M, Mora A, Ashby P (2002). Essential role of PDK1 in regulating cell size and development in mice. EMBO J.

[31] Li J, Liu S, Zhou H, Qu L, Yang J (2014). starBase v2.0: decoding miRNA-ceRNA, miRNA-ncRNA and protein-RNA interaction networks from large-scale CLIP-Seq data. Nucleic Acids Res.

[32] Taylor B, DeCarolis P, Angeles C (2011). Frequent alterations and epigenetic silencing of differentiation pathway genes in structurally rearranged liposarcomas. Cancer Discov.

[33] Du Y, Liu Z, Gu L (2012). Characterization of human gastric carcinoma-related methylation of 9 miR CpG islands and repression of their expressions in vitro and in vivo. BMC Cancer.

[34] Rauhala H, Jalava S, Isotalo J (2010). miR-193b is an epigenetically regulated putative tumor suppressor in prostate cancer. Int J Cancer.

[35] Hang S, Wang X, Li H (2019). Triptolide inhibits viability and migration while promotes apoptosis in nephroblastoma cells by regulation of miR-193b-3p. Exp Mol Pathol.

[36] Li X, Rui B, Cao Y, Gong X, Li H (2020). Long non-coding RNA LINC00152 acts as a sponge of miRNA-193b-3p to promote tongue squamous cell carcinoma progression. Oncol Lett.

[37] Choi H, Kim H, Kim E (2015). An age-dependent alteration of the respiratory exchange ratio in the db/db mouse. Lab Anim Res.

[38] Esser K, Su W, Matveev S (2007). Voluntary wheel running ameliorates vascular smooth muscle hyper-contractility in type 2 diabetic db/db mice. Appl Physiol Nutr Metab.

[39] Mazzu Y, Hu Y, Soni R (2017). miR-193b-regulated signaling networks serve as tumor suppressors in Liposarcoma and promote Adipogenesis in adipose-derived stem cells. Cancer Res.

[40] Belarbi Y, Mejhert N, Lorente-Cebrián S (2015). MicroRNA-193b controls adiponectin production in human white adipose tissue. J Clin Endocrinol Metab.

[41] Arner E, Mejhert N, Kulyté A (2012). Adipose tissue microRNAs as regulators of CCL2 production in human obesity. Diabetes.

[42] Ju B, Nie Y, Yang X (2019). miR-193a/b-3p relieves hepatic fibrosis and restrains proliferation and activation of hepatic stellate cells. J Cell Mol Med.

[43] Camera D, Smiles W, Hawley J (2016). Exercise-induced skeletal muscle signaling pathways and human athletic performance. Free Radic Biol Med.

[44] Gan Z, Fu T, Kelly D, Vega R (2018). Skeletal muscle mitochondrial remodeling in exercise and diseases. Cell Res.

