# Supplementary material for: MicroRNA-193b impairs muscle growth in mouse models of type 2 diabetes by targeting the PDK1/Akt signalling pathway
Source: Diabetologia. 2021 Dec 16;65(3):563–81. doi: 10.1007/s00125-021-05616-y (PMC8803817; doi:10.1007/s00125-021-05616-y)
Supplement: Supplementary file 1 — (PDF 1539 kb) [file 125_2021_5616_MOESM1_ESM.pdf]

MicroRNA-193b impairs muscle growth in mouse models of type 2 diabetes by targeting the PDK1/Akt signalling pathway

Shu Yang[1,2] (ORCID: 0000-0002-4912-777X), Guangyan Yang[1], Han Wu[1,3], Lin Kang[1], Jiaqing Xiang[1], Peilin Zheng[1] (ORCID: 0000-0002-4712-4903), Shanhu Qiu[1], Zhen Liang[4] (ORCID: 0000-0003-4700-0203), Yan Lu[5], Lijing Jia[1]

[1]Department of Endocrinology, The Second Clinical Medical College, Jinan University (Shenzhen People's Hospital), Shenzhen, China

[2]Integrated Chinese and Western Medicine Postdoctoral Research Station, Jinan University, Guangzhou, China

[3]Department of Endocrinology, Key Laboratory of Endocrinology, National Health Commission, Peking Union Medical College Hospital, Peking Union Medical College and Chinese Academy of Medical Sciences, Beijing, China

[4]Department of Geriatrics, The Second Clinical Medical College, Jinan University (Shenzhen People's Hospital), Shenzhen, China

[5]Department of Endocrinology and Metabolism, Zhongshan Hospital, Fudan University, Shanghai, China

ESM Methods

Serum miR-193b analysis For analysis of miR levels in serum, an miR-193b primer was used. The miR-193b level in serum was normalised to cel-miR-39 (25 fmol/l single-stranded cel-miR-39 [synthesised by Invitrogen] was spiked into serum as an internal control, and the relative miR-193b level in tissues was normalised to the mRNA level of β-actin.

Histological analysis Muscle tissue was embedded in paraffin, and 5 μm thick serial

sections for staining were cut using a paraffin slicer. For muscle histology, the paraffin slicers were stained with Mayer's hematoxylin and eosin staining kit (Solarbio & Technology, Beijing, China).

Determination of re-fed serum glucose and serum insulin level For re-fed blood glucose, mice were fasted overnight and then the food was replaced for 2 h before the determination of serum glucose level. Serum insulin levels were determined using mouse insulin ELISA kit (catalogue no.: ab277390) obtained from Abcam (Cambridge, MA, USA) after treatment.

Glucose tolerance tests and insulin tolerance tests Glucose tolerance tests (GTTs) and insulin tolerance tests (ITTs) were performed in mice at 15 and 16 weeks of age as previous described, respectively [1]. Briefly, mice fasted for 6 hr and then were intraperitoneally injected with glucose (2 g glucose/kg body weight; catalogue no.: Y0001745, Sigma-Aldrich, St.Louis, MO, USA) for the GTT assay or with insulin (1 U insulin/kg body weight; Actrapid, Novo Nordisk, Denmark) for ITT assay. And then glucose levels were determined at 0, 15, 30, 60, 90 and 120 min after injection. During the treatment, the fasting blood glucose were determine with the blood sample withdrawn from mouse tail vein using the OneTouch glucometer and test strips (LifeScan, Milpitas, CA, USA).

Immunofluorescent staining for myofibre type Immunofluorescent staining was

conducted for myofibre type in gastrocnemius muscle from miR-139b AAV-treated C57 and sgmiR-193b AAV-treated *db/db* mice. In brief, gastrocnemius and TA muscles were embedded in paraffin, and 5 μm thick serial sections for staining were cut using a paraffin slicer. Paraffin sections were dewaxed with xylene and hydrated with gradient ethanol (100%, 95%, 80%, 70%, 50%). Then sections were placed in a box filled with citric acid antigen retrieval buffer (pH 6.0; catalogue no.: G1202-250ML; Servicebio, Wuhan, China) in a microwave oven for antigen retrieval. After washing in PBS (catalogue no.: G0002; Servicebio) for 5 minutes, sections blocked with 10% of goat serum in PBS for 1 h at room temperature. The sections incubated with the mixed primary antibody (see below) overnight at 4 ℃, and then the sections washed with PBS three times. Subsequently, the sections incubated with secondary antibodies (see below) for 50 min at room temperature in the dark. Rabbit anti-fast myosin skeletal heavy chain antibody (diluted 1:100 in PBS; catalogue no.: GB112130; Servicebio) and mouse anti-slow skeletal myosin heavy chain antibody (diluted 1:100 in PBS; catalogue no.: GB21303; Servicebio) were used as primary antibodies, whilst Cy3-labelled goat anti-rabbit IgG (catalogue no.: GB21303; Servicebio) and Alexa Fluor 488-labelled goat anti-mouse IgG (catalogue no.: GB25301; Servicebio)(both diluted 1:400) were used as secondary antibodies. DAPI (catalogue no.: P0131-25ml; Beyotime, Shanghai, China) was used to stain cell nuclei. Images were captured and processed with a Leica DMi8 automated microscope using (Leica, Weztlar, Germany) and the myofibre area was measured using ImageJ software (RRID: SCR_003070; version 1.51; NIH, Bethesda, MD, USA).

qPCR All reagents were obtained from Sigma-Aldrich, except where indicated. Total RNA was extracted from cells or tissue using RNAiso Plus. The sequences of oligonucleotide primers synthesised by Generay (Shanghai, China) are shown in ESM Table 1. The relative levels of mRNA expression were normalised to the loading control (actin) by Quantity One Software (Bio-Rad Laboratory, CA, USA).

Determination of mitochondrial DNA copy numbers Quantification of mitochondrial DNA (mtDNA) copy numbers was achieved by PCR as previous described [2]. Briefly, DNA was extracted from gastrocnemius using a DNeasy Blood and Tissue kit (catalogue no.: 69504, QIAGEN, Duesseldorf, Germany). Total DNA was subjected to qPCR using LightCycler 480 SYBR Green (Roche, Basel, Switzerland) according to the manufacturer's instructions. In brief, 1 μl total DNA (100 ng/ml) was mixed with 4 μl dH_2O and 5 μl PCR mix (LightCycler 480 SYBR Green and 0.5 μM of each primer). mtDNA or nuclear DNA was amplified using primers against *Cox-3* (forward, 5'-ttgaagctgtacaacctacc-3'; reverse, 5'-cctgcgattaaggcatgatg-3') or primers against *Act1* (forward, 5'-caccctgttcttttgactga-3'; reverse, 5'-cgtagaaggctggaacgttg-3'), respectively. Relative mtDNA copy number changes were calculated using the comparative ΔΔCt method [3] by determining Ct threshold values and using equations ΔΔCt = (Ct_*Cox-3* − Ct_*Act1*) at t(n) − (Ct_*Cox-3* − Ct_*Act1*) at t(0) and $2^{-\Delta\Delta Ct}$.

Measurement of glycogen and lactate Mouse skeletal muscle were performed by standard procedure using a glycogen assay kit (catalogue no.: ab65620) and lactated assay kit (catalogue no.: ab169558), respectively, obtained from Abcam (Cambridge, MA, USA).

Hanging test The mice were placed on a wire (length 100 cm, diameter 2 mm and height 40 cm from the ground). The wire was then rotated to place the mice in hanging state. The time for which the mouse could hold itself was recorded, then the average time for each group was compared with that of other groups. The tests were repeated three times for each mouse and each time with a 2 min rest period.

Locomotor activity Locomotor activity was performed using a computer-controlled, open-circuit system (Oxymax; Comprehensive Lab Animal Monitoring System; Columbus Instruments, Columbus, OH, USA). Mice were tested in individual clear chambers (20×10×12.5 cm) with a stainless steel elevated wire floor. Each chamber contained a sipper tube delivering water, a food tray connected to a balance, and 16 photobeams at 1.3-cm intervals situated in rows 3.3 and 7.3 cm above the floor to detect motor activity along the x-, y-, and z-axes, respectively.

Grip Strength Test A digital grip strength meter purchased from Columbus instrument was used to assess forelimb grip of mice. The mouse was lifted by its tail and held on to the forelimb lever. Mice were lifted by their tails, allowed to grasp the

forelimb pull bar, and pulled smoothly away from the transducer in the horizontal plane, and maximum force exerted on the transducer was recorded. This test was repeated for 3 sets, 10 times for each set, with a 15-minute rest between each set. And record the maximum effort of each mouse at each attempt and assign it to the maximum grip.

Maximal oxygen consumption test Mice were placed on the treadmill (Jiangsu SANS Biological Technology Co. Ltd, China), and the shock grid was activated (0° incline entire experiment). The treadmill speeds were then increased until exhaustion as shown in the figure 3 and 5, respectively. Exhaustion (endpoint for treadmill cessation) was defined as the point at which mice maintained continuous contact with the shock grid for 5 s.

Western blotting For western blotting, protein lysates from C2C12 cells or tissues obtained from mice (TA or gastrocnemius) were prepared. Immunoblotting-specific antibodies used were as follows: β-actin (catalogue no.: ab213262), S6K (catalogue no.: ab32359), mTOR (catalogue no.: ab25880), p-mTOR (Ser2448; catalogue no.: ab109268), Akt (catalogue no.: ab8805), p-Akt (Ser473; catalogue no.: ab81283) and myosin heavy chain 7B (Myh; catalogue no.: ab172967) (all from abcam); and p-S6K (Thr389) (catalogue no.: 9205S), PDK1 (catalogue no.: 3062S), AMPKα (catalogue no.: 2532S), and p-AMPKα (catalogue no.: 50081S) (all from Cell Signaling Technology, MA, USA). Diluted fresh primary antibody (1:1000) or anti-β-actin

antibody (1:5000) in PBS containing 1% (wt/vol.) fresh dry fat-free milk (Solarbio, Beijing, China), and 1:5000 diluted fresh horseradish peroxidase (HRP)-conjugated anti-rabbit (catalogue no.: sc-2004; Santa Cruz Biotechnology) or mouse (catalogue no.: sc-2005; Santa Cruz Biotechnology) IgG in PBS containing 1% (wt/vol.) fresh dry fat-free milk. The membrane was incubated with fresh primary antibodies overnight at 4 ℃, and then incubated with secondary antibodies for 1 h at room temperature. Densitometry analysis was performed using Quantity One Software (Bio-Rad Laboratory, CA, USA) and quantified to the loading control, β-actin.

Immunofluorescent staining of C2C12 cells Immunofluorescence staining of C2C12 cells was performed in the order of fixation, permeation, and incubation with primary antibodies against, for Myh immunostaining, Myh (catalogue no.: ab172967; Abcam) antibodies were used. DAPI (catalogue no.: P0131-25ml; Beyotime) was used to stain cell nuclei. The images were captured and processed with a Leica DMi8 automated microscope using (Leica).

Immunostaining of C2C12 cells was performed in the order of fixation, permeation, and incubation with primary antibodies against, for Myh immunostaining, Myh (catalogue no.: ab172967; Abcam) antibodies were used. The images were captured and processed with a Leica DMi8 automated microscope using (Leica, Wetzlar, Germany), and the myofibre area was measured using ImageJ software.

Dual-luciferase reporter assay C2C12 cells were transfected with 3' UTR luciferase reporter constructs (3' UTR-PDK1, 3' UTR-PDK1-mutant), miRNA (Ctrl RNA [catalogue no.: sc-36869, Santa Cruz Biotechnology] or miR-193b-3p) and Renilla luciferase using Lipofectamine 3000, according to manufacturer's instructions (Invitrogen). After 48h of transfection, luciferase activity of cells was measured using a Dual Luciferase Assay Kit (catalogue no.: E1910, Promega, Madison, WI, USA) and microplate reader (catalogue no.: H1, BioTeK, Biotek Winooski, Vermont, USA). Luciferase activity of each group was calculated and graphed. Renilla luciferase was used to normalize the value of firefly luciferase.

Resuspension of Pdk1 siRNA *Pdk1* siRNA is a pool of 3 target-specific 19-25 nt siRNAs designed to knock down gene expression. Each vial contains 3.3 nmol of lyophilized siRNA. Resuspend lyophilized siRNA duplex in 330 µl of the RNAse-free water provided. Resuspension of the siRNA duplex in 330 µl of RNAse-free water makes a 10 µM solution in a 10 µM Tris-HCl, pH 8.0, 20 mM NaCl, 1 mM EDTA buffered solution.

ESM Table 1 The sequences of primers for qPCR analysis

Gene	Forward	Backward
mus-Actc1	CTGGATTCTGGCGATGGTGTA	CGGACAATTTCACGTTCAGCA
mus-Pgc1α	TTCATCTGAGTATGGAGTCGCT	GGGGGTGAAACCACTTTTGTAA
mus-Tnnc1	GCGGTAGAACAGTTGACAGAG	CCAGCTCCTTGGTGCTGAT
mus-Pgc1β	TCCTGTAAAAGCCCGGAGTAT	GCTCTGGTAGGGGCAGTGA
mus-Tnnt1	CCTGTGGTGCCTCCTTTGATT	TGCGGTCTTTTAGTGCAATGAG
mus-Tnni1	ATGCCGGAAGTTGAGAGGAAA	TCCGAGAGGTAACGCACCTT
mus-MyHC IIa	AAGTGACTGTGAAAACAGAAGCA	GCAGCCATTTGTAAGGGTTGAC
mus-MyHC IIb	TTGAAAAGACGAAGCAGCGAC	AGAGAGCGGGACTCCTTCTG

mus-Tnni2	AGAGTGTGATGCTCCAGATAGC	AGCAACGTCGATCTTCGCA
mus-Tnnc2	GAGGCCAGGTCCTACCTCAG	GGTGCCCAACTCTTTAACGCT
mus-Baf60c	CCCGAGTCCCAGGCTTACA	GCTTTCGCTTTTGCTTCATGG
mus-Tbx15	CTCCGTTGAAGCCTTGATCGG	AGACGCCAGGTCAGTGTGA
mus-β-actin	GGCTGTATTCCCCTCCATCG	CCAGTTGGTAACAATGCCATGT

ESM Table 2 The basic clinical characteristics of participants

basic clinical characteristics	healthy control	Patients with type 2 diabetes
Number of participants	20	20
Age	54.75±9.77	55.05±8.97
Sex	9:11 (male:female)	12:8 (male:female)
ethnicity	Chinese	Chinese

ESM Table 3 The basic clinical characteristics of patients with type 2 diabetes

basic clinical characteristics	Patients with type 2 diabetes (n=20)
FBG (mmol/l)	8.01±2.083
TG (mmol/l)	3.41±3.37
HbA1c (mmol/mol)	80.03±28.9
CP (nmol/l)	0.72±0.47
Body weight (kg)	64.13±13.0
BMI (kg/m^2)	23.96±2.65
Fat mass (kg)	18.63±4.60
Lean mass (kg)	24.81±6.45
Fat mass/body weight (%)	29.28±5.87
Lean mass/body weight (%)	38.45±3.98

ESM figures

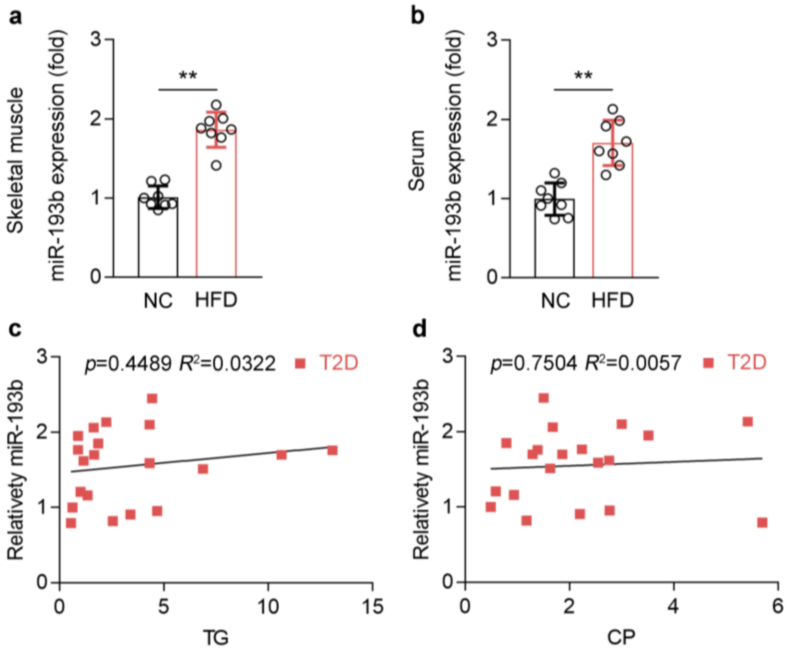

ESM Figure 1. MiR-193b increases in HFD mice and is no significant correlation with TG and CP. (a, b) The miR-193b level was determined in both skeletal muscle (**a**) and serum (**b**) in both normal control (NC) mice and 16-week HFD fed mice, n=8. **(c, d)** Serum samples collected from type 2 diabetes (T2D) were determined the following parameters: miR-193b, TG (mmol/l) (**c**) and CP (ng/ml) (**d**), (human, n=20). Red squares = Type 2 diabetes. ***p* < 0.01 vs. NC by unpaired Student's t test.

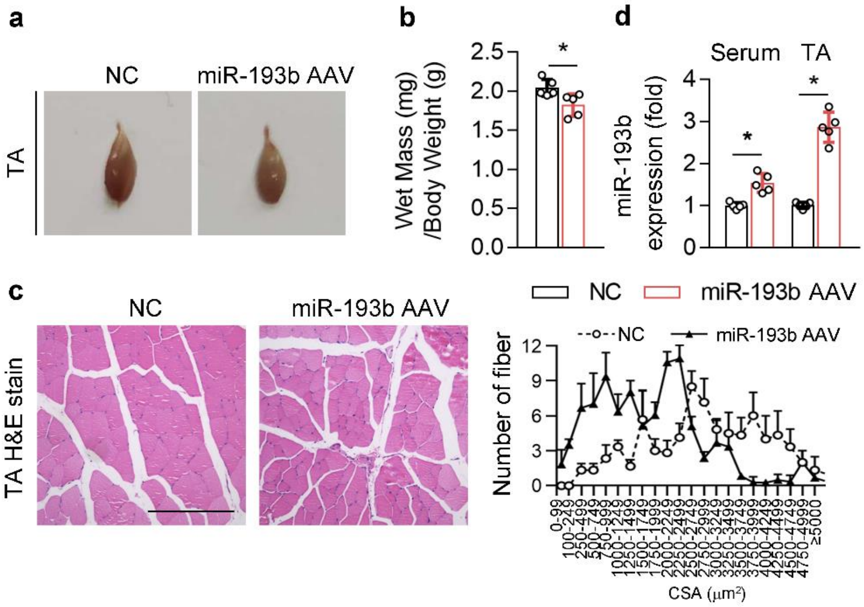

ESM Figure 2. In situ injection of AAV9-HSA-miR-193b induces muscle atrophy. AAV9-Hsa-miR-193b (miR-193b AAV) or AAV9-Hsa-*gfp* (NC) was injected into the tibialis anterior (TA) at three independent points, and skeletal muscle were harvested

8 weeks later after injection. **(a)** Photographs of isolated muscle types from mice. **(b)** Weights of TA from mice, n=5. **(c)** Histology of TA as determined by hematoxylin and eosin staining in both NC group and AdmiR-193b group (scale bar: 250 µm), n=3. **(d)** The qRT-PCR determined miR-193b level in serum and TA, n=5. **p* < 0.05 vs. NC by unpaired Student's t test.

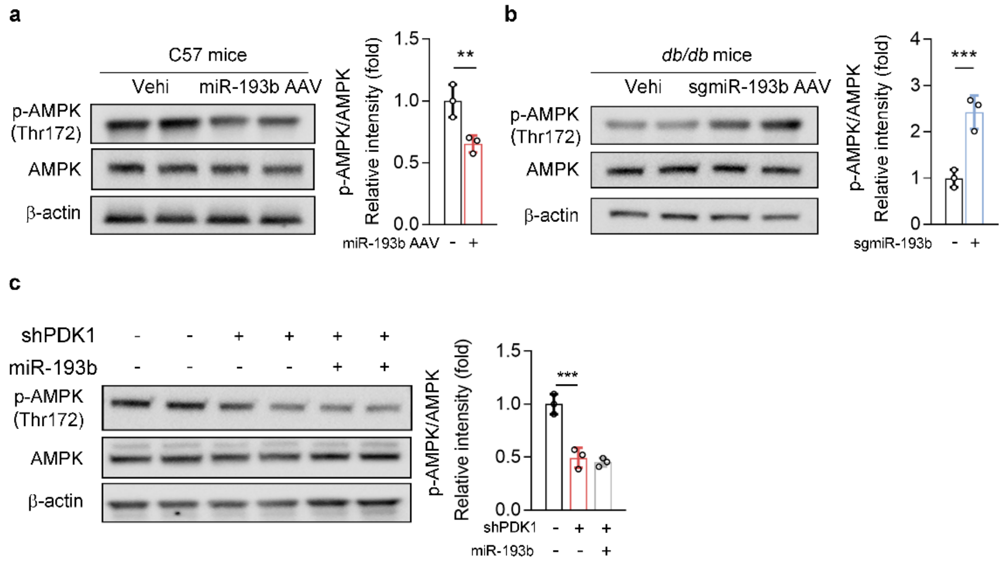

ESM Figure 3. MiR-193b inhibits AMPK phosphorylation rely on PDK1. (a-c) AMPK and its phosphorylation determined by western blot in skeletal muscle of C57 or *db/db* mice as indicated in figure, and quantification of the relative levels of p-AMPK proteins was shown in the right panel, n=3. ***p* < 0.01 vs. NC, ****p* < 0.001 vs. NC by unpaired Student's t test in panel a, b. ****p* < 0.001 vs. NC by one-way ANOVA with Bonferroni correction in panel c.

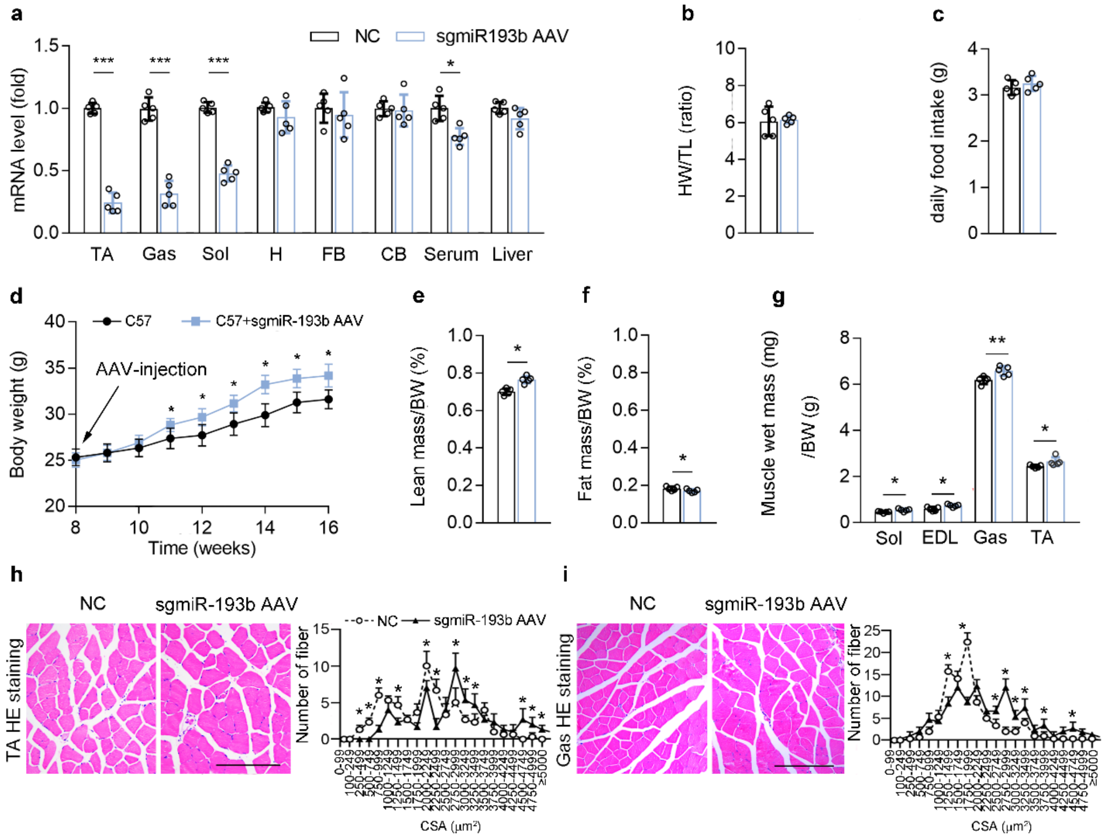

ESM Figure 4. Skeletal muscles miR-193b-deficient increases muscles mass in C57 mice. C57 mice were injected with AAV-Hsa-gfp (NC) or AAV-Hsa-sgmiR-193b (sgmiR-193b AAV) through tail vein at 8 weeks of age. **(a)** The qRT-PCR determined miR-193b level in TA, Gas, Sol, H, FB, CB, serum and liver, n=5. **(b)** The ratio of heart weight and tibia length, n=5. **(c)** Daily food intake, n=5. **(d)** Body weight changes over time, *p < 0.05 vs. NC at the same time point, n=5. **(e, f)** Body mass measured by a MRI (16 weeks of age, n=5). **(g)** Weights of four muscle types from NC or AAV-Hsa-sgmiR-193b injected mice for 8 weeks, n=5. **(h, i)** Histology of TA (**h**) and Gas (**i**) muscles as determined by hematoxylin and eosin staining in C57 mice and C57 mice+sgmiR-193b (scale bar: 250 μm), and quantitative results were shown on the right panel, n=5. *p < 0.05 vs. NC, **p < 0.01 vs. NC, ***p < 0.001 vs. NC by unpaired Student's t test.

Yang *et al.* ESM Figure 5

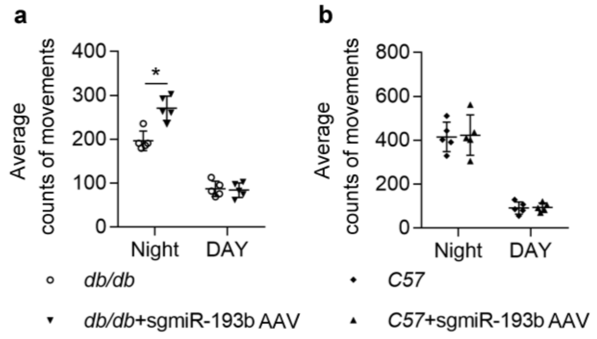

ESM Figure 5. locomotor activity of *db/db* or C57 mice. (**a, b**) Mice were tested in individual clear chambers with a stainless steel elevated wire floor. Each chamber contained a sipper tube delivering water, a food tray connected to a balance and photobeams to detect activities, n=5. *p < 0.05 vs. NC by unpaired Student's t test.

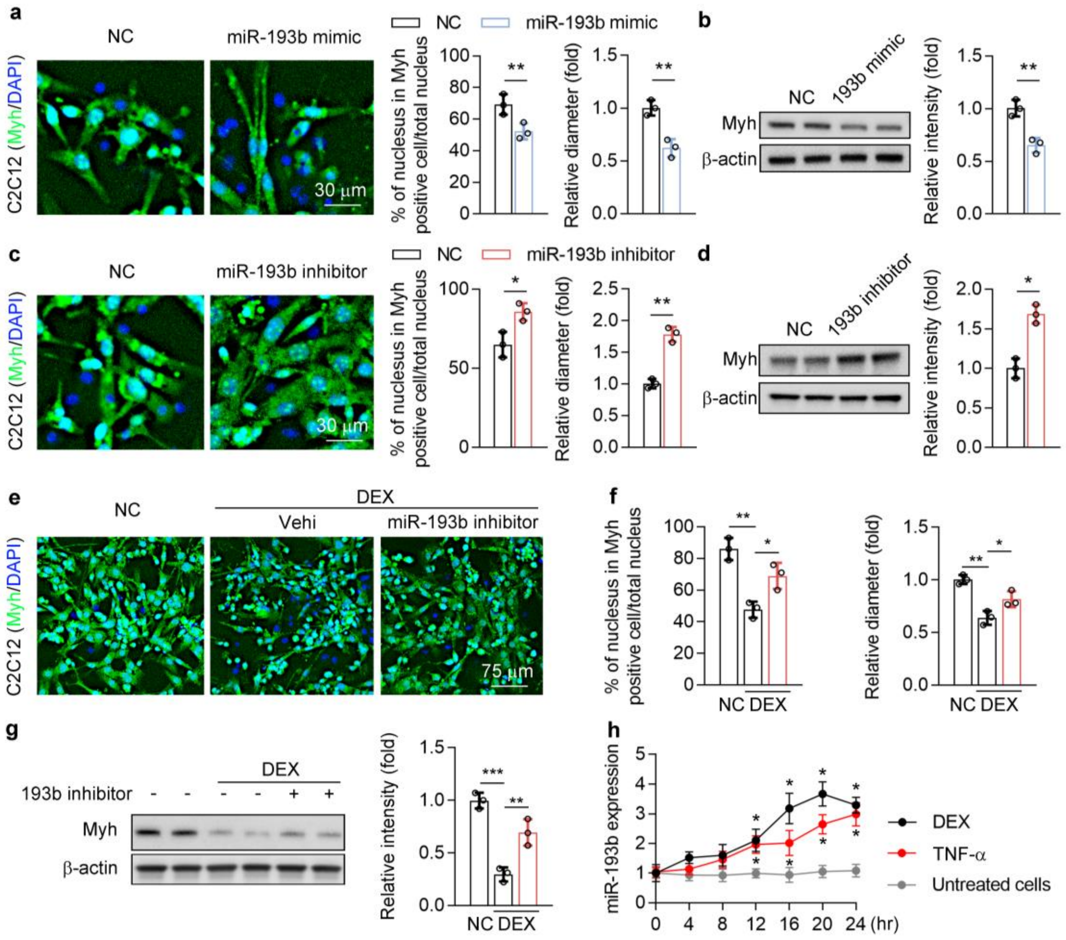

ESM Figure 6. Myotube formation is inhibited by miR-193b in C2C12 cells. (a-d) C2C12 cells treated with normal differentiation medium for 1 day and treated with

vehicle, miR-193b mimic (40 nM), miR-193b inhibitor (100 nM) for another 2 days in differentiation medium (scale bar, 30 μm) (NC means cells treated with nontargeting negative control RNA). C2C12 cells were scored as percentage of nucleus in Myh positive cell/total nucleus per field were shown in the middle panel, and quantification of Myh-positive myotube diameter per field were shown in the right panel, n=3 (a, c). Western blot analysis of Myh expression in C2C12 cells, the quantification of the relative levels of Myh proteins was shown in the right panel, n=3 (b, d). **p* < 0.01, ***p* < 0.001 by unpaired Student's t test. **(e-g)** Immunostaining for Myh expression in C2C12 cells treated with normal differentiation medium for 1 day and treated with dexamethasone (DEX, 10 μM) alone or DEX and miR-193b inhibitor combination for another 1 days in differentiation medium (scale bar, 75 μm) (NC means cell treated with nontargeting negative control RNA). Quantification of myotube formation in panel e, and Myh positive cell/total nucleus and Myh-positive myotube diameter, n=3 (f). Western blot analysis of Myh expression in C2C12 cells, the quantification of the relative levels of Myh proteins was shown in the right panel, n=3 (g). **p* < 0.05, ***p* < 0.01, ****p* < 0.001, by one-way ANOVA with Bonferroni correction. **(h)** C2C12 cells treated with normal differentiation medium for 1 day, and then C2C12 cells treated with DEX (10 μM) or TNF-α (20 ng/ml) for another 1 days in differentiation medium, qRT-PCR analysis was used to detect the expression of miR-193b, n=5 (h). **p* < 0.05 vs. untreated cells at the same time point by unpaired Student's t test.

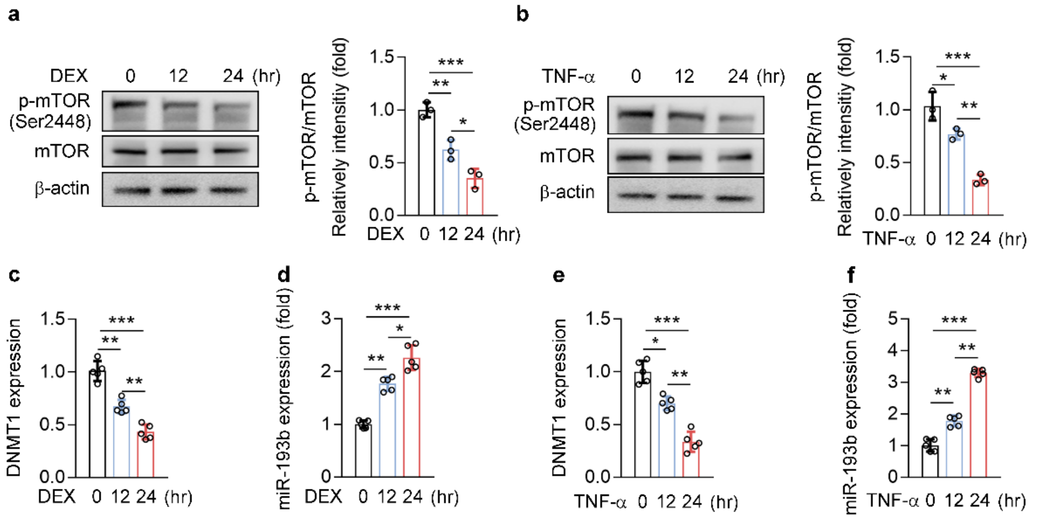

ESM Figure 7. TNF-α and DEX treatment inhibits mTOR phosphorylation and DNMT1 expression, leading to the upregulation of miR-193b. (a, b) Western blot analysis for phosphorylation of mTOR in C2C12 cells treated with normal differentiation medium for 1 day and treated with DEX (10 μM) or TNF-α (20 ng/mL) as indicated for another 1 days in differentiation medium, the quantification of the

relative levels of p-mTOR proteins was shown in the right panel, n=3. **(c-f)** The mRNA level of *Dnmt1* (**c,e**) and miR-193b (**d, f**) expression were determined by qRT-PCR, n=5. *p < 0.05, **p < 0.01, ***p < 0.001, by one-way ANOVA with Bonferroni correction.

ESM References

[1] Yang S, Ma C, Wu H, et al. (2020) Tectorigenin attenuates diabetic nephropathy by improving vascular endothelium dysfunction through activating AdipoR1/2 pathway. Pharmacol Res 153: 104678. 10.1016/j.phrs.2020.104678

[2] Medeiros T, Thomas R, Ghillebert R, Graef M (2018) Autophagy balances mtDNA synthesis and degradation by DNA polymerase POLG during starvation. The Journal of cell biology 217(5): 1601-1611. 10.1083/jcb.201801168

[3] Livak K, Schmittgen T (2001) Analysis of relative gene expression data using real-time quantitative PCR and the 2(-Delta Delta C(T)) Method. Methods (San Diego, Calif) 25(4): 402-408. 10.1006/meth.2001.1262
